# Neointimal hyperplasia and vascular restenosis: from molecular mechanisms to therapeutic interventions

**DOI:** 10.1186/s43556-026-00477-6

**Published:** 2026-05-25

**Authors:** Lingyan Yi, Tingting Chen, Yulin Zhou, Peile Zhu, Qingyu Zhu, Yuting Shao, Wenjuan Yao

**Affiliations:** 1 School of Pharmacy, Nantong University, 9 Seyuan Road, Nantong, Jiangsu 226019 China; 2 Xinglin College, Nantong University, 9 Seyuan Road, Nantong, Jiangsu 226019 China; 3 Department of Pharmacology, School of Pharmacy, Nantong University, 9 Seyuan Road, Nantong, Jiangsu 226019 China

**Keywords:** Vascular restenosis, Neointimal hyperplasia, Phenotypic transformation, Re-endothelialization, Molecular targets

## Abstract

Vascular restenosis, a pathological recurrence of lumen narrowing following interventions, remains a major limitation to the long-term success of vascular procedures. Its development is centrally driven by neointimal hyperplasia, a process orchestrated by endothelial injury, phenotypic switching of vascular smooth muscle cells (VSMCs) from a contractile to a synthetic state, and a coordinated inflammatory response. Despite advancements, the molecular mechanisms are not fully elucidated, and specific, effective pharmacotherapies are still lacking. This review systematically delineates the pathophysiology, focusing on these three core elements, and provides a comprehensive landscape of the complex signaling networks and molecular targets. We extensively cover protein-based regulators—including pro-proliferative factors (e.g., LSD1, FGF10), protective mediators (e.g., CGRP, A20), dual-action molecules with isoform-specific or context-dependent effects (e.g., KLFs, HDACs), endothelial repair targets (e.g., VEGF), and molecules that coordinately target both VSMCs and endothelial cells (ECs) (e.g., PERK, METTL3). We place significant focus on non-coding RNAs, particularly microRNAs (miRNAs) like miR-221/222, which fine-tune multiple targets in both VSMCs and ECs, offering unique precision. We critically evaluate the therapeutic significance and clinical translation potential, while addressing formidable challenges: functional duality within protein families (e.g., KLFs, HDACs), difficulties in cell-specific delivery and stability for miRNA therapies, and a narrow therapeutic window. Additionally, we highlight the emerging role of the vascular adventitia as a key source of regulatory signals (e.g., FGF10). By integrating insights from molecular mechanisms to therapeutic interventions, this work serves as a valuable reference for identifying novel strategies to combat neointima formation and vascular restenosis.

## Introduction

Vascular restenosis is a common pathological phenomenon following cardiovascular interventional therapies [1], significantly impacting long-term patient prognosis and quality of life. Since the widespread adoption of balloon angioplasty and drug-eluting stents (DES), the mechanisms underlying restenosis have remained a focal point of cardiovascular research. Although DES have markedly reduced restenosis rates, challenges such as late-stage restenosis and in-stent thrombosis persist, suggesting that current therapeutic strategies are still limited in their ability to modulate the underlying molecular mechanisms. Consequently, a thorough understanding of the molecular mechanisms governing vascular restenosis and the identification of precise, safe interventional targets have become central tasks in contemporary research.

Despite considerable progress in restenosis research in recent years [2–4], with a proliferation of related molecular targets, a systematic synthesis and integration of this knowledge is lacking [5]. Existing studies often focus on individual molecules or pathways, whereas the nature of vascular restenosis is a multifactorial, multicellular pathological process driven by intricate, interwoven signaling networks. In the context of clinical translation, many targets prove difficult to apply in practical therapy due to a lack of specificity, potential side effects, or poorly understood mechanisms. Therefore, a comprehensive and systematic review of the currently known key molecular targets is urgently needed. Such a review must clarify their roles, mechanisms of action, and potential clinical application prospects in the initiation and progression of vascular restenosis, thereby providing clear direction and reference for subsequent basic research and clinical translation.

This review focuses on three aspects: the pathophysiology of vascular restenosis, its molecular mechanisms, and the molecular targets that promote or inhibit restenosis. For the first time, this review presents a "molecular target atlas," systematically integrating the diverse range of molecular targets implicated in vascular restenosis research. This atlas encompasses targets that promote proliferation/migration, inhibit proliferation/migration, exhibit dual functions, and those related to endothelial repair. Furthermore, this review highlights the dual roles and potential therapeutic value of emerging regulatory molecules, such as protein kinase RNA-like ER kinase (PERK), N^6^-methyladenosine methyltransferase (METTL3), and miR-22, in restenosis. This demonstrates a shifting research paradigm from "single-target inhibition" towards a more holistic "regulation of the microenvironmental network".

## The pathophysiology of neointima formation and vascular restenosis

Research indicates that neointima formation is a key contributor to the vascular restenosis. Following vascular injury, vascular smooth muscle cells (VSMCs) transform from the contractile to the synthetic phenotype and acquire the ability to proliferate and migrate. These activated VSMCs migrate to the subintima, proliferate extensively, and secrete extracellular matrix components, culminating in neointima formation and subsequent luminal narrowing. Additionally, inflammatory cell infiltration and the release of inflammatory mediators cause endothelial cell (EC) damage and dysfunction, which in turn triggers abnormal VSMC proliferation and migration, exacerbating neointima formation and vascular stenosis. Therefore, identifying key molecular mechanisms and targets involved in neointima formation provides a vital foundation for developing novel therapeutic strategies against vascular restenosis. Targeting and inhibiting neointimal hyperplasia offers a promising approach to effectively prevent and treat restenosis, thereby reducing cardiovascular complications and improving patient prognosis and quality of life. We will focus on elucidating the pathophysiological mechanisms underlying vascular intimal thickening and restenosis through the lens of three core elements.

### Endothelial injury and dysfunction—the initiating event

Endothelial injury is a key initiating factor for neointimal hyperplasia. Both clinical and experimental evidence highlight that maintaining endothelial integrity plays a crucial role in mitigating neointimal hyperplasia. For instance, the eSVS mesh, which wraps around saphenous vein grafts, prevents expansive endothelial injury and subsequent neointimal hyperplasia by maintaining structural stability [6]. Genetically modified endothelial progenitor cells (EPCs) can promote re-endothelialization of injured arteries, thereby effectively inhibiting neointimal hyperplasia [7]. Cellular communication network factor 5‌ (CCN5), a member of the matricellular protein family, effectively alleviates neointima formation and in-stent restenosis (ISR) by promoting EC repair, and CCN5-coated stents may hold promise for preventing ISR after percutaneous coronary intervention (PCI) [8]. In a study involving 89 chronic kidney disease (CKD) patients undergoing arteriovenous fistula (AVF) creation, 80% presented with pre-existing endothelial alterations, including medial hypertrophy (51 cases) and significant intimal hyperplasia (19 cases), suggesting that endothelial dysfunction precedes overt neointimal hyperplasia [9]. Notably, in an in vitro model, indoxyl sulfate (IS)-induced endothelial microparticles (EMPs) were found to exacerbate neointimal hyperplasia by activating the transforming growth factor-β (TGF-β) signaling pathway [10]. A variety of potential circulating biomarkers related to endothelial function have been identified for assessing endothelial function [11]. Emerging evidence suggests that some of these markers may indeed hold predictive value.

Research on endothelial dysfunction primarily revolves around oxidative stress, mitochondrial dysfunction, and inflammation [12–16]. Oxidative stress is associated with decreased endothelial nitric oxide synthase (eNOS) activity and increased reactive oxygen species (ROS) generation, with NADPH oxidase and uncoupled eNOS identified as major sources [17]. High glucose increases ROS production in ECs by approximately 50%, while resveratrol mitigates this effect via the phosphatidylinositol 3-kinase (PI3K)/protein kinase B (AKT)/nuclear factor erythroid 2-related factor 2 (Nrf2) signaling pathway, and cinnamic acid targets peroxisome proliferator-activated receptor (PPAR) δ through the Nrf2/heme oxygenase 1 (HO-1) and AKT/eNOS pathways, alleviating oxidative stress and endothelial dysfunction [18–20]. Studies on chronic venous disease (CVD) further emphasize the role of ROS: superoxide anion radicals reduce nitric oxide (NO) bioavailability, leading to vasoconstriction and hypoxia [15].

Both clinical trials and basic research have confirmed that mitochondrial dysfunction is a key factor in the development of oxidative stress-induced vascular endothelial injury [21, 22]. Mitochondria are important sites of ROS production, and mitochondrial ROS (mtROS) are a critical factor in vascular endothelial dysfunction. Clinical trials have shown that treatment with the mitochondria-targeted antioxidant MitoQ for 6 weeks improves endothelial function and reduces mtROS levels [23, 24]. Recently, Eun-Mi Kim et al. developed a mitochondria-targeting SS31-conjugated liposome, which was shown in vitro to reduce EC oxidative stress and protect endothelium from oxidative damage [25]. Furthermore, mitochondrial oxidative stress in ECs leads to impaired vascular barrier function [26].

Additionally, inflammatory pathways play a crucial role: following endothelial injury, activation of coagulation pathways, downregulation of thrombomodulin, and the action of increased von Willebrand factor result in platelet adhesion and aggregation, release of inflammatory mediators, and progression to a prothrombotic state, ultimately promoting vascular pathology [27–30]. The cytokine storm triggered by COVID-19 activates ECs, increasing the expression of vascular cell adhesion molecule-1 (VCAM-1) and intercellular adhesion molecule-1 (ICAM-1) by up to 30% [31]. The NOD-like receptor protein 3 (NLRP3) inflammasome pathway is considered a marker of endothelial inflammation and dysfunction [32]. Age-related reduction in vagal output induces a systemic proinflammatory state and may mediate endothelial hyperpermeability and barrier dysfunction via the angiopoietin-Tie2 signaling axis, adrenomedullin, and vascular endothelial cadherin [33, 34]. Resolvin E1 (RvE1), derived from eicosapentaenoic acid, has been reported to promote endothelial repair by inhibiting neutrophil infiltration and promoting M2 macrophage polarization, thereby reducing neointimal hyperplasia in a murine arterial injury model [35]. Hepatocyte growth factor (HGF) gene transfer enhances endothelial regeneration by improving eNOS activity and reduces VSMC proliferation to attenuate neointimal hyperplasia [36]. Conversely, interferon-induced protein 35 (IFI35) inhibits the proliferation and migration of ECs by suppressing the nuclear factor kappa-B (NF-κB)/p65 pathway, thereby delaying endothelial reformation and promoting neointimal hyperplasia [37]. MicroRNAs (miRNAs) have been shown to play roles in endothelial dysfunction. Studies have found that miR-146a, miR-21, and miR-126 regulate the NF-κB signaling pathway and EC proliferation, and their dysregulation promotes atherosclerosis [38]. Inhibition of miR-155 alleviates endothelial inflammation and promotes endothelial regeneration, and the use of a miR-155 inhibitor-coated stent resulted in a 20% reduction in restenosis rate [39].

Current research has consolidated a central pathophysiological triad in endothelial dysfunction: oxidative stress, mitochondrial dysfunction, and inflammation. A major conceptual advance is the recognition of mitochondria as the critical hub, where mtROS are identified as a primary driver. This has led to novel therapeutic strategies, such as mitochondria-targeted antioxidants (e.g., MitoQ, SS31-conjugated liposomes), which directly address this source of injury. In parallel, endothelial inflammation and dysregulated endothelial repair are critical progression factors. Novel findings show that specific miRNAs (e.g., miR-155) can directly inhibit EC regeneration, thereby creating a permissive environment for neointimal hyperplasia. This mechanistic understanding is being translated into next-generation interventions like miR-155 inhibitor-eluting devices, which aim to actively promote endothelial healing while preventing restenosis.

### VSMC phenotypic modulation—the core executor

While endothelial injury is the trigger, the subsequent pathological response is driven by the underlying VSMCs. The transition of VSMCs from a contractile to a synthetic phenotype is a central event in neointima formation, with their phenotypic plasticity being a current research focus [40–43]. Vascular injury stimulates VSMCs to undergo phenotypic switching into a dedifferentiated cell type, known as the synthetic VSMC, which possesses enhanced migratory and proliferative capacities and participates in vascular repair processes [44]. Platelet-derived growth factor-BB (PDGF-BB) is a potent stimulator: VSMCs treated with PDGF-BB exhibit a 35% higher proliferative capacity compared to controls, an effect mediated by extracellular signal-regulated kinase 1/2 (ERK1/2) [45]. Single-cell sequencing and lineage tracing studies reveal that VSMCs originate from adventitial stem/progenitor cells and exist in multiple phenotypes. These phenotypic VSMCs downregulate the expression of contractile proteins such as α-smooth muscle actin (α-SMA) and calponin 1, while acquiring specific markers and functional characteristics resembling osteoblasts, fibroblasts, macrophages, and mesenchymal cells, thereby mediating vascular neointima formation [44, 46]. Induced pluripotent stem cell (iPSC)-derived VSMCs provide a novel tool for studying this type of vascular disease, as these cells can recapitulate VSMC phenotypic switching [47].

The molecular mechanisms underlying VSMC phenotypic modulation involve the precise regulation of multiple signaling pathways and gene expression networks. Recent studies have found that protein arginine methyltransferase 5 (PRMT5) promotes VSMC phenotypic change and contributes to vascular intimal thickening by modulating histone methylation and acetylation levels, thereby hindering the expression of contractile markers mediated by serum response factor (SRF)/myocardin (myocd) complexes [48]. The ETS transcription factor ELK1/staphylococcal nuclease domain-containing protein 1 (SND1)/SRF signaling represents a novel pathway that promotes a proliferative VSMC phenotype and neointimal hyperplasia in vascular injury, predisposing vessels to pathological remodeling [49]. The mechanosensitive Piezo1 channel drives VSMC proliferation and migration and promotes neointimal hyperplasia by activating yes-associated protein (YAP) and transcriptional co-activator with PDZ-binding motif (TAZ) via Ca^2^⁺ and its downstream effectors calmodulin kinase II and calcineurin [50]. Furthermore, the reprogramming of glycolysis and fatty acid synthesis also cooperatively promotes vascular injury-induced VSMC dedifferentiation and neointimal formation [51]. Some proteins and pathways play important roles in maintaining the VSMC contractile phenotype. For instance, blood vessel epicardial substance (Bves) maintains the contractile phenotype of VSMCs and subsequently inhibits neointima formation via a dual-specificity protein phosphatase 1 (Dusp1)-dependent mitogen-activated protein kinase p38 (p38MAPK)/ERK1/2 signaling pathway [52]. Bone morphogenetic protein (BMP) EC precursor-derived regulator (BMPER) binds to insulin-like growth factor-binding protein 4 (IGF-BP4), thereby modulating the IGF signaling pathway and promoting the transition of VSMCs towards a contractile phenotype [53]. Nexilin (NExN) mediates VSMC phenotypic transformation via the endoplasmic reticulum (ER) stress and kruppel-like factor 4 (KLF4) signaling pathway. VSMC-specific NExN deficiency promotes VSMC phenotypic switching and exacerbates neointimal hyperplasia following vascular injury in mice [54]. Additionally, serum vasostatin-2 attenuates vascular injury-induced neointimal hyperplasia in mice and inhibits VSMC proliferation in vitro through the angiotensin-converting enzyme 2 (ACE2)/nuclear receptor subfamily 1 group D member 1 (NR1D1)/growth arrest-specific 1‌ (Gas1) pathway. Decreased levels of vasostatin-2 are associated with coronary restenosis in patients undergoing coronary angioplasty [55].

Mitochondrial metabolism and dynamics also modulate VSMC behavior [56, 57]. Dynamin-like protein 1 (DLP1)-mediated mitochondrial fission is essential for VSMC migration, and inhibition of DLP1 reduces PDGF-induced lamellipodia formation and ROS production [58]. High-density lipoprotein (HDL) helps maintain VSMC mitochondrial homeostasis by suppressing the upregulation of dynamin-related protein 1 (Drp1)—a protein involved in mitochondrial fission—reducing ROS generation, and preventing loss of mitochondrial membrane potential [59]. Rictor, a component of mitochondrial mammalian target of rapamycin complex 2 (mTORC2), has been shown to promote VSMC proliferation via TGF-β signaling, and Rictor-knockout mice exhibit a 25% reduction in restenosis [60]. Our previous research also confirmed that the mitochondrial protein A-kinase anchor protein 1 (AKAP1) improves protein kinase A (PKA)-mediated phosphorylation of Drp1 at Ser637, inhibits mitochondrial fission, and thereby protects VSMCs from phenotypic modulation, preventing neointima formation [61]. Mitochondria further regulate VSMC phenotype and associated cardiovascular diseases spatiotemporally through interactions with the cytoskeleton [62]. Therefore, maintaining mitochondrial homeostasis within VSMCs may represent an effective approach to alleviating pathological vascular remodeling [63, 64].

In addition, miRNAs play a key role in regulating VSMC behavior [65]. For example, miR-34c targets stem cell factor (SCF), reducing VSMC proliferation by 30% [66], while miR-140-3p improves VSMC behavior by inhibiting Ras homolog family member A (RhoA)/Rho-associated coiled-coil containing protein kinase (ROCK) signaling, thereby suppressing proliferation and migration [67]. Clinically, DES targeting VSMC proliferation, such as paclitaxel-coated stents, have demonstrated significant efficacy in reducing restenosis [68]. Combination therapies show even stronger effects: in vitro studies indicate that stents coated with prednisolone (PD) and sirolimus (SRL) can reduce restenosis rates by 46.8%, where PD suppresses inflammation and SRL inhibits VSMC proliferation [69]. Moreover, certain natural compounds also exhibit promising anti-proliferative and anti-restenotic effects on VSMCs. For instance, capsaicin, a natural compound, inhibits VSMC proliferation by 40% through modulation of calcium signaling [70]. Magnolia kobus DC (MO) improves VSMC phenotypic switching and intimal thickening by regulating ferroptosis and ROS generation [71]. Ginkgolic acid (GA) inhibits VSMC proliferation, migration, and vascular restenosis by modulating transmembrane protein tectonic 1 (TCTN1) to influence cell cycle progression and cytoskeletal rearrangement [72]. Colchicine partially reverses VSMC phenotypic switching by regulating the expression of myocd, a key regulator of the contractile phenotype in VSMCs [73].

These mechanistic understandings are paving the way for next-generation interventions. These range from miRNA-based strategies (e.g., miR-34c, miR-140-3p) and novel natural compounds (e.g., capsaicin, GA) that target specific pathways, to advanced device-based approaches like multi-drug coated stents. Future directions lie in leveraging single-cell omics to decipher pathological VSMC subsets in humans and developing cell-state-specific therapeutics that precisely inhibit pathological switching while preserving or restoring contractile function, offering promising strategies for preventing restenosis and pathological vascular remodeling.

### Inflammation and immune cell recruitment — the coordinators

Critically, the phenotypic switching and proliferation of VSMCs do not occur in a vacuum; they are powerfully coordinated and amplified by local inflammatory responses. Inflammatory responses mediated by cytokines and immune cells also serve as driving forces for neointimal hyperplasia [74–76]. Early studies revealed that under diabetic conditions, fibroblast-specific protein 1 (FSP-1) activates ROCK1 in ECs, increasing intimal permeability and inflammatory cell infiltration, thereby exacerbating vascular restenosis [77]. Moreover, inhibiting the NF-κB signaling pathway in ECs adjacent to synthetic VSMCs effectively suppresses neointima formation [78]. Recent research has identified the innate immune sensor stimulator of interferon genes (STING) as a promoter of VSMC proliferation, migration, and intimal thickening through NF-κB signaling [79]. NF-κB-inducing kinase (NIK) has been recognized as a key driver of vascular inflammatory responses and lumen narrowing [80]. Meanwhile, miRNAs such as miR-155, miR-146a, and miR-21 function as core regulators of inflammatory pathways; inhibition of miR-155 reduces NF-κB activation by 40% [81]. Additionally, macrophages and pro-inflammatory cytokines, including interleukin 6 (IL-6), IL-1β, and tumor necrosis factor α (TNF-α), can modulate VSMC phenotype and intimal thickening *in vitro* [82–85]. N-myc downstream-regulated gene 1 (NDRG1) is involved in recognizing and regulating endothelial inflammation, thrombotic responses, and vascular remodeling. Inhibiting NDRG1 may thus represent a novel therapeutic strategy for treating inflammatory vascular diseases such as atherosclerotic thrombosis and restenosis [86].

Interferon regulatory factor 4 (IRF4) has been found to play an important protective role in the development of neointimal hyperplasia by regulating macrophage polarization [87]. The potent anti-inflammatory mediator RvE1 inhibits vascular neutrophil infiltration in a mouse femoral artery injury model, promotes macrophage polarization toward an M2-like phenotype, and suppresses T-cell trafficking by reducing RANTES secretion [88]. Another pro-resolving lipid mediator, resolvin D1 (RvD1), and its derivatives have also been shown to attenuate neointimal hyperplasia following acute arterial injury in rats [89]. Furthermore, studies indicate that a ketogenic diet inhibits VSMC proliferation and migration by suppressing oxidative stress and inflammatory responses, ultimately reducing neointimal hyperplasia [75]. Dehydroepiandrosterone can inhibit the proliferation and inflammatory responses of VSMCs and the vascular intima by regulating the miR-486a-3p/NLRP3 signaling axis [90]. The selective vitamin D receptor activator—paricalcitol—significantly downregulates the expression of growth differentiation factor 15 (GDF-15), cluster of differentiation 74 (CD74), and NIK, thereby inhibiting inflammation and vascular restenosis [91]. In summary, controlling inflammatory responses represents an effective strategy for counteracting intimal hyperplasia [92–94]. Combination therapies targeting both inflammation and VSMC proliferation show promising potential [95–97]. For instance, nano-aggregates constructed from pH-responsive endosome-escaping polymers and a MAPK-activated protein kinase 2 (MK2) peptide inhibitor effectively block vascular inflammatory and VSMC migratory signaling pathways, significantly reducing the extent of intimal hyperplasia [98]. Vascular stents immobilized with the anti-inflammatory chemerin 15 peptide inhibit inflammatory cell adhesion, modulate macrophage polarization, promote inflammation resolution and rapid re-endothelialization, while simultaneously suppressing VSMC proliferation and migration to prevent restenosis [99]. These findings underscore the synergistic effects of anti-inflammatory and anti-proliferative agents in mitigating neointimal hyperplasia. Therapeutically, the field is moving beyond broad immunosuppression toward targeted immunomodulation and multi-pathway integration. Future prospects lie in precisely targeting pathological immune cell subsets and harnessing resolution biology, offering a refined approach to prevent restenosis by restoring vascular immune homeostasis.

## Molecular mechanisms: decoding signaling networks

Based on a clear understanding of the pathological process of vascular restenosis, a more fundamental scientific question emerges: what intricate molecular networks drive and regulate the occurrence and progression of these pathological events? An in-depth decoding of the aforementioned pathological processes must rely on a systematic analysis of the underlying signaling networks. In recent years, the research perspective has shifted from individual molecules or pathways to a holistic understanding of multi-dimensional and dynamic regulatory networks. This includes exploring how the synergy and antagonism of key signaling pathways (such as PI3K/AKT, MAPK, and PDGF/PDGF receptor (PDGFR) signaling) precisely direct cell fate, and how epigenetic regulations (e.g., histone modifications, DNA/RNA methylation) "write" and stabilize aberrant cellular phenotypes at the genomic level. These mechanisms do not operate in a linear sequence but are interwoven into a highly interconnected regulatory network that collectively determines whether the direction of vascular repair after injury leads to physiological healing or pathological stenosis.

### Key signaling pathways

#### PI3K/AKT

The PI3K/AKT signaling pathway serves as a downstream convergence point for multiple growth factors and cytokines, functioning as a central hub regulating cell proliferation, survival, and metabolic reprogramming. It is a pivotal driver in the processes of vascular intimal thickening and restenosis. Following vascular injury, this pathway is potently activated by factors such as PDGF and angiotensin II (Ang II). In rat carotid artery balloon injury models, its activity can increase by 3- to fivefold and remain elevated for at least 14 days [100, 101]. In type 1 diabetic rat arterial injury models, insulin also promotes intimal hyperplasia via the PI3K/AKT pathway [102]. Activated AKT drives the phenotypic switch and proliferation of VSMCs directly through phosphorylation of downstream targets such as protein kinase B (PKB) [103]. Conversely, inhibition of AKT signaling activates glycogen synthase kinase-3β (GSK-3β), leading to mitochondrial depolarization, increased ROS production, activation of redox-sensitive plasma membrane voltage-gated potassium channels, and decreased intracellular calcium concentration, thereby significantly suppressing VSMC proliferation and promoting apoptosis, ultimately attenuating vascular remodeling [104]. Activation of the PI3K/AKT pathway promotes cell cycle progression not only in normal, non-transformed SMCs in vitro but also in SMCs following vascular injury in vivo, thereby contributing to the development of restenosis and atherosclerosis [105]. Furthermore, this signaling cascade drives VSMC proliferation via activation of the mTORC1. A combined therapeutic approach using the PI3K inhibitor LY294002 along with the mTOR inhibitor rapamycin effectively suppresses leptin-induced neointimal hyperplasia [106, 107]. Rapamycin-eluting stents significantly inhibit neointimal hyperplasia and local vascular inflammation in porcine coronary arteries without inducing hepatorenal toxicity or cardiomyocyte damage, demonstrating favorable efficacy and safety profiles [108]. The chemokine CCL11 (eotaxin-1), specifically secreted by chemotactic fibroblasts, activates the PI3K/AKT pathway in SMCs in a paracrine manner, thereby promoting their proliferation, migration, and intimal hyperplasia [109]. Mechanical stretch applied to the vascular wall during stent implantation may contribute to in-stent restenosis by activating the AKT signaling pathway, and the PI3K inhibitor wortmannin can mitigate the extent of neointimal hyperplasia following stent placement [110]. These findings collectively establish the PI3K/AKT signaling pathway as a critical therapeutic target [111].

#### PDGF/PDGFR

Upon vascular injury, activated platelets and inflammatory cells release PDGF-BB, which binds to the highly expressed PDGFR-β on the surface of VSMCs. This binding triggers receptor dimerization and autophosphorylation, activating multiple downstream signaling cascades such as PI3K/AKT and β-catenin, thereby strongly driving the phenotypic switch of VSMCs from the contractile to the synthetic type. This transition is a key step in intimal thickening, characterized by downregulation of contractile markers (e.g., SMC) and upregulation of proliferation markers (e.g., proliferating cell nuclear antigen, PCNA) [112–114]. Activated PDGF/PDGFR signaling promotes cell cycle progression by upregulating cyclin D1 and downregulating cyclin-dependent kinase (CDK) inhibitors such as p27, enabling cells to cross the G1/S checkpoint and enter the proliferation cycle [115, 116]. Simultaneously, this pathway remodels the cytoskeleton by activating Rho family small GTPases (e.g., Rac1 and Cdc42) and upregulates the expression of matrix metalloproteinases (MMPs) (e.g., MMP-2 and MMP-9) to degrade the extracellular matrix barrier, collectively facilitating the migration of VSMCs from the media to the intima [117–119]. Notably, PDGF signaling also forms a positive feedback loop with the local inflammatory microenvironment: it can induce vascular cells to secrete chemokines such as monocyte chemoattractant protein-1 (MCP-1), recruiting more inflammatory cells that in turn serve as a sustained source of PDGF [120, 121]. Furthermore, PDGF downregulates soluble guanylyl cyclase (sGC) expression through PI3K and Rac1, thereby altering Notch ligand signaling, indicating crosstalk between PDGF and NO/sGC pathways in human VSMCs. This interaction may represent a potential target for intervening in neointimal hyperplasia [122]. Preclinical studies have confirmed that targeting this pathway with PDGFR tyrosine kinase inhibitors (e.g., imatinib) or neutralizing antibodies effectively inhibits abnormal proliferation and migration of VSMCs and reduces neointimal area in rat carotid artery balloon injury models [123, 124]. Although systemic administration faces challenges due to the broad physiological functions of this pathway, it remains a highly promising target for local therapies (e.g., drug-coated balloons) and is a key strategy for intervening in vascular remodeling and preventing restenosis.

#### MAPK/ERK

The MAPK/ERK signaling pathway is highly conserved and has been demonstrated to regulate a variety of biological processes, serving as a core signaling cascade that mediates neointimal hyperplasia and restenosis following vascular injury [125]. Upon vascular injury, stimuli such as PDGF, Ang II, and mechanical stretch rapidly activate this pathway, leading to increased phosphorylation levels of ERK1/2 and promoting the proliferation and migration of VSMCs [126–128]. The MAPK/ERK pathway is also involved in mediating endothelin-1-induced VSMC contraction and proliferation. Endothelin-1 induces ERK1/2 activation in a concentration- and time-dependent manner, with the activation peaking at 10 min post-stimulation. This endothelin-1-induced ERK1/2 activation is associated with enhanced intracellular activity of PKC, PKA, and PI3K [129]. Studies have further shown that high glucose-induced expression of VCAM-1 in ECs is dependent on the MAPK signaling pathway, thereby contributing to endothelial injury [130]. Inhibition or blockade of MAPK/ERK signaling exerts anti-proliferative effects on VSMCs and attenuates vascular neointimal thickening [131, 132]. Some natural compounds, such as Farrerol, maintain the contractile phenotype of VSMCs by blocking MAPK/ERK signaling, effectively suppressing VSMC phenotypic switching and balloon injury-induced neointima formation [133]. Inhibitors targeting the MAPK/ERK pathway, delivered via local strategies such as drug-coated balloons or novel nanocarriers, represent a potential therapeutic approach for preventing in-stent restenosis.

#### Wnt/β-catenin

The Wnt/β-catenin signaling pathway, an evolutionarily conserved pathway crucial for embryonic vascular development, is aberrantly reactivated following vascular injury in adults, becoming one of the pathological mechanisms driving neointimal hyperplasia and restenosis. Studies have revealed that after vascular endothelial injury, exposed SMCs, aggregated platelets, and inflammatory cells release various Wnt ligands (such as Wnt4 and Wnt3a). These ligands bind to Frizzled receptors and disrupt the "destruction complex" composed of Axin, APC, and GSK-3β, thereby preventing the phosphorylation and ubiquitin-mediated degradation of β-catenin. The stabilized and accumulated β-catenin translocates into the nucleus, where it forms a complex with transcription factors of the TCF/LEF family, initiating the transcriptional program of a series of pro-proliferative and pro-fibrotic genes, including c-Myc, Cyclin D1, and fibronectin. This process directly drives the phenotypic switch of VSMCs from a quiescent, contractile state to a highly proliferative, synthetic state [134, 135]. Notably, this process is more pronounced in patients with metabolic diseases such as hyperlipidemia. Hyperlipidemia can strongly upregulate the mRNA expression levels of Wnt3a, β-catenin, and Cyclin D1, further amplifying β-catenin signaling and leading to more severe vascular remodeling [136]. The Wnt/β-catenin pathway can also activate the expression of its downstream target genes, MMP2 and MMP7, thereby promoting VSMC migration [137]. Research has found that the deubiquitinating enzyme Cezanne promotes VSMC proliferation by targeting β-catenin for deubiquitination, thereby regulating the expression of CCN family member 1 (CCN1) [138].

The role of the Wnt/β-catenin pathway extends far beyond promoting cell proliferation. Recent studies emphasize that sustained activation of Wnt/β-catenin signaling induces pathological transdifferentiation of VSMCs, enabling them to acquire osteoblast- or chondrocyte-like characteristics. This is a critical step leading to vascular ectopic calcification and intimal hyperplasia, and a key feature of late-stage restenosis in the DES era [139, 140]. Furthermore, this pathway does not operate in isolation; it engages in extensive crosstalk with other signaling networks such as Notch, TGF-β, and PI3K/AKT, forming complex positive feedback loops. For instance, β-catenin can upregulate the expression of the Notch ligand Jagged-1, while the activated Notch intracellular domain can, in turn, stabilize β-catenin. This synergistic interaction collectively locks VSMCs into an undifferentiated, progenitor-like state with high proliferative potential, significantly contributing to the long-term growth and stabilization of the neointima [141]. The TGF-β pathway promotes VSMC proliferation by inducing the secretion of canonical Wnt proteins, which further stabilizes β-catenin [142]. Wnt3a and TGF-β can cooperatively induce β-catenin-dependent responses. Wnt3a enhances TGF-β signaling by promoting the phosphorylation of TGF-β-activated kinase 1 (TAK1) and upregulating the synthesis and secretion of IL-11 [143]. The PI3K/AKT pathway suppresses GSK-3β activity, thereby reducing β-catenin degradation, which leads to sustained activation of the Wnt/β-catenin pathway, a mechanism involved in regulating processes like myocardial differentiation [144]. Recent research has uncovered a functional interaction between the Wnt/β-catenin and sphingosine-1-phosphate (S1P) signaling pathways. The carboxyl-terminal domain of β-catenin mediates the expression of the sphingosine-1-phosphate receptor 1 (S1PR1), and this interaction is indispensable during neointima formation [145].

Preclinical studies have shown that using β-catenin inhibitors (such as ICG-001 and PKF118-310) or specifically knocking out β-catenin can downregulate genes promoting neointima formation, including MMP2, MMP9, sphingosine kinase 1 (SPHK1), and S1PR1, while upregulating genes inhibiting neointima formation, such as Jagged1 and gap junction alpha-1 protein (GJα1). Therefore, inhibiting β-catenin holds promise as a therapeutic strategy to reduce abnormal VSMC accumulation and alleviate vascular obstruction [146]. However, due to the broad physiological functions of the Wnt pathway in many tissues, systemic inhibition faces challenges such as skeletal toxicity and other adverse effects. Consequently, therapeutic strategies targeting Wnt/β-catenin signaling are increasingly focused on developing vascular-localized, specific delivery systems. This approach aims to precisely intervene in pathological vascular remodeling while minimizing systemic side effects, offering new avenues for overcoming the challenge of restenosis.

#### NF-κB

The NF-κB signaling pathway, serving as a core transcriptional regulatory hub for inflammatory responses, plays a pivotal role in intimal thickening and restenosis following vascular injury [147]. Upon vascular endothelial injury, hemodynamic changes, oxidative stress, and inflammatory cell infiltration rapidly activate the IκB kinase complex, leading to phosphorylation and subsequent ubiquitin-dependent degradation of the NF-κB inhibitory protein IκBα. This releases NF-κB (predominantly the p50/p65 heterodimer), which translocates into the nucleus and initiates the transcription of hundreds of downstream target genes [148–150]. These genes include pro-inflammatory cytokines (e.g., TNF-α, IL-1β), chemokines (e.g., MCP-1), adhesion molecules (e.g., VCAM-1, ICAM-1), and MMPs, collectively establishing a positively amplified inflammatory microenvironment [151]. Notably, NF-κB activation occurs not only in infiltrating macrophages [152, 153] but is also critically involved in VSMCs. Studies have found that activation of the NF-κB pathway can mediate VSMC phenotypic switching in a cell-autonomous manner and contribute to neointima formation after vascular injury [154].

Recent research has unveiled more sophisticated regulatory mechanisms of this pathway. At the epigenetic level, histone deacetylase 3 (HDAC3) binds to NF-κB p65 and deacetylates multiple lysine residues, thereby activating NF-κB and exacerbating the inflammatory response [155, 156]. Consequently, HDAC inhibition has frequently demonstrated significant anti-inflammatory effects in both clinical and preclinical settings. For instance, inhibition of HDAC9 attenuates NF-κB pathway activation, exerting a protective effect in oxidized low‑density lipoprotein (ox-LDL)-induced human umbilical vein ECs (HUVECs) injury [157]. Conversely, SIRT1 inhibits NF-κB expression through its deacetylase activity, thereby alleviating coronary artery spasm mediated by the myosin light-chain kinase (MLCK)/myosin regulatory light chain 2 (MLC2)/endothelin-1 (ET-1) signaling axis [158]. This indicates that different types of HDACs play distinct roles in regulating NF-κB signaling. At the metabolic level, lipid metabolism disorders can, via the NF-κB pathway, increase rat vascular cell apoptosis rates by 24%, proliferation rates by 18%, and EC damage by 33%, consequently promoting atherosclerosis and accelerating the rate of vascular aging by 27% [159]. Furthermore, mechanical signals are also involved in regulation: four weeks after coronary stent implantation in swine, NF-κB mRNA and protein levels were significantly elevated, triggering pronounced vascular inflammation and fibrosis [160]. This pathway also promotes NLRP3 gene transcription and inflammasome activation via the nuclear translocation of NF-κB p65 and p50 heterodimers, and mediates the expression of the pyroptosis key protein gasdermin D (GSDMD), thereby contributing to vascular remodeling and in-stent restenosis [161–163].

Local delivery of neomycin sulfate (applied to the adventitial surface of injured vessels in a Pluronic acid gel formulation) counteracts neointimal hyperplasia by inhibiting the activation of MAPK and NF-κB [164]. Clinical trials have also shown that local transfection or stent coating with NF-κB-specific inhibitors (e.g., using decoy oligonucleotides to competitively bind p65) can significantly suppress neointima formation and restenosis, an approach with substantial clinical application potential [165, 166]. Certain traditional Chinese medicines alleviate vascular restenosis by targeting NF-κB signaling. For example, the compound formula Fufang Zhenzhu Jiangzhi Capsule (FTZ) reduces restenosis by inhibiting NF-κB activity and the expression of inflammatory factors within atherosclerotic plaques, positioning it as a potential therapeutic agent for restenosis [167]. Recently, miRNAs have also been recognized for their significant role in combating vascular restenosis. For instance, tail vein injection of miR-7-5p inhibits VSMC migration and intimal hyperplasia after vascular injury by suppressing NF-κB p65 activity [168]. MiR-520c-3p regulates the proliferation, apoptosis, and adhesion of ECs by targeting the NF-κB signaling pathway, playing an important role in inhibiting endothelial injury [169]. Although NF-κB is indispensable for immune homeostasis, serving as a core component of innate and adaptive immune responses, it remains a highly promising target for anti-restenosis therapy. Utilizing nano-carriers for targeted delivery or developing small molecules that selectively inhibit specific IκB kinase subunits holds the potential to effectively curb pathological vascular remodeling while preserving physiological immune surveillance.

#### Hippo/YAP/TAZ

The primary function of the Hippo-YAP/TAZ signaling pathway is to regulate cell proliferation, differentiation, and migration within organs. Early studies in rodent models demonstrated its involvement in the pathogenesis of vascular restenosis [170]. While the Hippo-YAP/TAZ pathway contributes to embryonic neovascularization, its role in physiological angiogenesis during adulthood appears limited. However, it plays a crucial part in pathological vascular remodeling [171, 172]. Unidirectional shear stress activates integrins on ECs and promotes their interaction with Gα13. This subsequently inhibits RhoA, leading to YAP phosphorylation and inactivation. Inactivated YAP/TAZ signaling suppresses the c-Jun N-terminal kinase (JNK) pathway and downregulates pro-inflammatory gene expression, thereby reducing monocyte adhesion and infiltration, which ultimately delays atherosclerosis progression. Conversely, disturbed atherosclerotic flow exerts opposing effects [173]. In spontaneously hypertensive rats (SHRs), YAP exhibits elevated nuclear expression alongside reduced cytoplasmic levels of both YAP and TAZ. This increased nuclear translocation of YAP/TAZ contributes to hypertensive vascular remodeling by upregulating forkhead box M1 (Foxm1) expression [174]. Furthermore, the Hippo-YAP/TAZ pathway regulates endothelial function. Ox-LDL treatment silences YAP expression by enabling miR-496 to bind to its 3' untranslated region (3' UTR). This prevents YAP from entering the nucleus and functioning as a transcriptional co-activator, ultimately inducing endothelial dysfunction [175].

Recent research has unveiled complex crosstalk between this pathway and other signaling networks. YAP/TAZ engage in synergistic interactions with the TGF-β/Smad pathway. TGF-β induces YAP binding to Smad2, an interaction essential for YAP nuclear translocation. Conversely, YAP and TAZ can bind Smad2/3, forming complexes that sequester Smads in the cytoplasm, thereby modulating their nucleocytoplasmic shuttling [176]. Additionally, vascular endothelial growth factor (VEGF) stimulation triggers cytoskeletal reorganization, promoting YAP/TAZ activation and nuclear entry. This regulates the expression of cytoskeleton-associated proteins, influencing cell motility and inducing endothelial proliferation, migration, and angiogenesis [176]. The Hippo-YAP/TAZ pathway is also closely linked to cell proliferation signals. In VSMCs, the YAP/TAZ-transcriptional enhanced associate domain (TEAD) complex increases the mRNA expression of pro-proliferative genes such as CCN1, connective tissue growth factor (CTGF), c-myc, and TGF-β2 [177]. YAP mediates Ang II-AT1 receptor signaling-induced VSMC proliferation, migration, and vascular remodeling [178]. Semaphorin 3G (Sema3G) inhibits large tumor suppressor kinase 1 (LATS1) and activates YAP via Nrp2/PlexinA1. Activated YAP directly regulates cyclin D1 and cyclin E1 expression, driving VSMCs through the G1/S checkpoint and promoting proliferation [179]. Conversely, downregulating YAP with sorafenib significantly upregulates contractile proteins in VSMCs and inhibits cyclin D expression [180].

Emerging evidence indicates that metabolic factors, including glucose, fatty acids, and hormones, regulate YAP and TAZ activity. In turn, YAP and TAZ participate in metabolic control by promoting glycolysis, lipid synthesis, and glutaminolysis, positioning them as key nodes coordinating nutrient availability with cell growth and tissue homeostasis [181]. For instance, the YAP/TEAD1 complex binds to the 6‑phosphofructo‑2‑kinase/fructose‑2,6‑biphosphatase 3 (PFKFB3) promoter, enhancing its transcriptional activity and thereby promoting VSMC calcification and EC glycolysis [182, 183]. A recent study found that in a rodent model of intimal hyperplasia, reduced O-GlcNAcylation of YAP weakens its liquid–liquid phase separation (LLPS). This impedes YAP binding to the signal transducer and activator of transcription 3 (STAT3) and reduces YAP nuclear translocation, consequently promoting neointimal hyperplasia [184].

Preclinical intervention studies provide compelling evidence for targeting this pathway. Verteporfin, which inhibits YAP by blocking its interaction with TEAD DNA-binding proteins, reduces the expression of the pathological marker thrombospondin-1 in vitro and attenuates intimal hyperplasia in vivo saphenous vein arterialization model in pigs [185]. Furthermore, a YAP-specific inhibitor (verteporfin)-eluting stent significantly suppresses restenosis and alleviates cerebral watershed infarction in a rabbit carotid artery injury model [186]. Beyond their lipid-lowering effects, statins have recently been shown to inhibit YAP nuclear localization by inducing its phosphorylation, cytoplasmic retention, and degradation, representing a potential non-canonical mechanism contributing to their anti-restenotic effects [187].

It is important to note that YAP plays a vital role in maintaining adult tissue homeostasis, and its complete inhibition could lead to vascular dysfunction. For example, VSMC-specific YAP/TAZ knockout mice spontaneously develop abdominal aortic aneurysms characterized by disorganized elastic fibers, VSMC apoptosis, proteoglycan accumulation, and immune cell infiltration, with pathology later extending to smaller arteries [188]. Therefore, future therapeutic strategies should focus on fine-tuning, rather than completely abolishing, YAP/TAZ activity. Recent advances in optogenetics and targeted nanocarrier delivery systems offer promising avenues for achieving spatiotemporally precise modulation of YAP signaling within injured vasculature [189].

### Epigenetic regulation

Epigenetic regulation serves as a critical molecular bridge linking environmental stimuli to persistent changes in gene expression, playing a pivotal role in intimal hyperplasia and restenosis following vascular injury. Its core mechanisms involve DNA methylation, histone modifications, and an intricate network of non-coding RNAs [190]. These studies have profoundly elucidated how vascular injury "locks" SMCs into a pathological phenotype through epigenetic mechanisms, enabling them to maintain sustained proliferative and inflammatory activity even after the initial stimulus subsides.

At the level of DNA methylation, vascular injury induces a comprehensive remodeling of the genome-wide methylation landscape, which represents an early event in the phenotypic switching of VSMCs [191]. Research indicates that by day 30 post-injury, neointimal cells exhibit a methylation profile more closely resembling that of progenitor cells than that of resident VSMCs [192]. DNA demethylation, achieved through the reduction of global 5-methylcytosine levels and hypermethylation of the TET2 (ten-eleven translocation 2) promoter, restores the enrichment of 5-hydroxymethylcytosine in the promoter region of myocd, thereby inhibiting VSMC dedifferentiation and vascular remodeling [193]. In the context of angioplasty-induced restenosis, the expression of DNA methyltransferase 1 (DNMT1) is upregulated, leading to hypermethylation of CpG islands in the promoter regions of VSMC contractile genes, such as SMA and smooth muscle 22α (SM22α). This results in their transcriptional silencing and mediates VSMC phenotypic switching [194]. Although DNMT1 is a potential therapeutic target for restenosis, inhibiting DNMT1 alone can lead to compensatory upregulation of DNMT3a, which may offset the loss of DNMT1 function. Therefore, concurrent inhibition of both DNMT1 and DNMT3a may be a necessary strategy for effectively preventing restenosis [195]. The DNA demethylation mediated by the TET family is also implicated: TET2 expression and activity are downregulated in a mouse model of aortic transplant vasculopathy. VSMC-specific knockout of TET2 suppresses the expression of key contraction-related genes such as SRF, while concurrently upregulating KLF4 transcription levels, leading to VSMC phenotypic switching, apoptosis, and medial thinning. Conversely, enhancing TET2 enzymatic activity with high-dose ascorbic acid (VitC) or locally overexpressing TET2 can restore contractile gene expression and reverse transplant vasculopathy-induced VSMC apoptosis and intimal thickening in a TET2-dependent manner [196, 197].

The complex dynamics of histone modifications constitute another core layer of epigenetic regulation. Histone-modifying enzymes play crucial roles in this process. For instance, deacetylase HDAC3 regulates endothelial-mesenchymal transition through post-translational modifications involving deacetylation and decrotonylation, thereby exacerbating intimal hyperplasia [198]. Alternative splicing of HDAC7 promotes VSMC proliferation and neointima formation by modulating β-catenin nuclear translocation; notably, the unspliced isoform (HDAC7u) binds to β-catenin and retains it in the cytoplasm, reducing its transcriptional activity [199]. Vascular injury can inhibit the expression and activity of the histone acetyltransferase p300 in the media while inducing elevated levels of CREB-binding protein (CBP) and histone acetylation, which consequently suppresses contractile protein expression, promotes cell migration, and leads to severe intimal hyperplasia [200]. Studies in mouse vascular injury models have confirmed that HDAC inhibition reduces neointima formation and decreases cyclin D1 expression [201, 202]. Histone methylation is equally critical [203]: the activity of methyltransferase EZH_2_ is significantly elevated in EC inflammation and atherosclerosis, and inhibiting EZH_2_ activity or silencing its expression proves effective in alleviating endothelial dysfunction and atherosclerosis [204]. Lysine-specific demethylase 1 A (KDM1A) exerts an inhibitory effect on BMP-2 activity, thereby promoting the phenotypic switch of VSMCs from a contractile to a synthetic state, neointima formation, and collagen deposition [205]. Furthermore, members of the Jumonji family of demethylases, key players in histone lysine demethylation, suppress H3K36 histone methylation levels, consequently promoting VSMC proliferation, migration, and vascular intimal thickening [206]. Emerging research has also identified histone lactylation as a novel mechanism linking metabolism and epigenetic control. This modification enhances the transcription of pyruvate dehydrogenase kinase 1, thereby accelerating lactate production in VSMCs and altering their metabolic and phenotypic profiles [207].

The network of non-coding RNAs forms precise regulatory circuits with epigenetic modifiers. Long non-coding RNAs (LncRNAs), such as SNHG12, can competitively bind to miR-15b-5p, thereby relieving its inhibitory effect on the endogenous target gene MYLK and promoting VSMC apoptosis [208]. Circular RNAs, like circLMF1, are significantly upregulated in PDGF-BB-induced human aortic VSMCs (HA-VSMCs). They regulate the expression of VEGFA or fibroblast growth factor 1 (FGF1) by sponging miR-125a-3p, consequently promoting cell cycle progression and migration [209]. The inherent reversibility of these epigenetic modifications renders them exceptionally attractive therapeutic targets, opening new dimensions for the prevention and treatment of restenosis.

## Molecular target atlas of vascular restenosis and its application prospects

It is well-established that the phenotypic switch of VSMCs from contractile to synthetic plays a pivotal role in neointima formation [210–212]. Consequently, most mechanistic studies focus on VSMC phenotypic modulation to identify effective intervention targets [48, 213]. Simultaneously, the imbalance between EC injury and repair profoundly influences neointimal development. Re-endothelialization during the repair process is crucial for restoring vascular integrity and inhibiting neointima formation [214–216]. Endothelial-derived factors such as NO [217–220], VEGF [221–223], and IL-6 [224–226] also intricately regulate VSMC behavior [227, 228]. This review will proceed to systematically summarize key molecular targets closely associated with vascular restenosis and neointima formation, specifically focusing on targets within VSMCs and ECs (Tables 1 and 2).

**Table 1:**
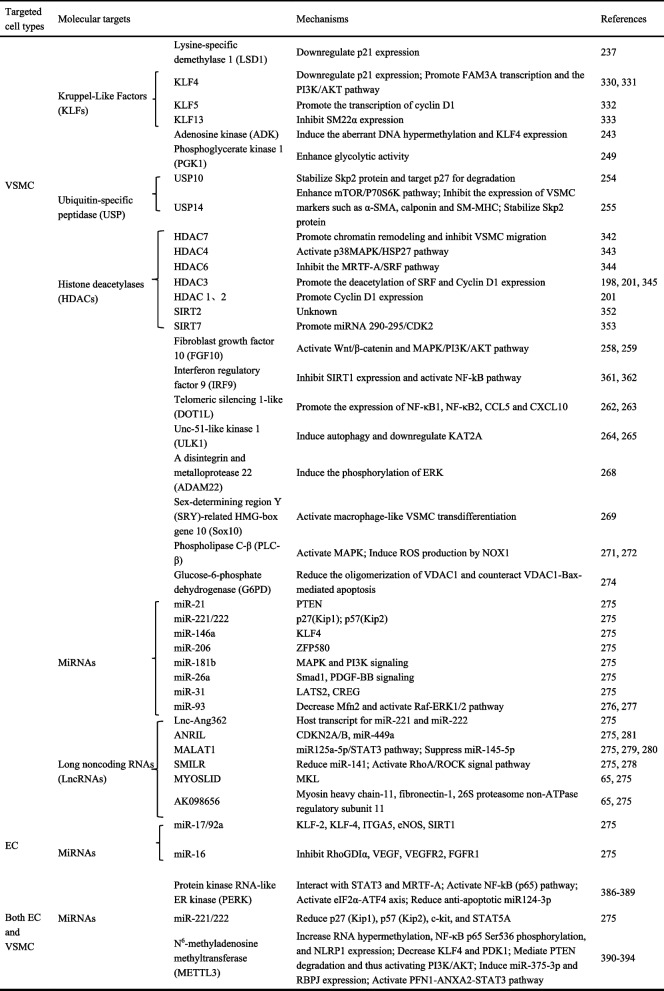
Targets for promoting neointima formation and vascular restenosis

**Table 2:**
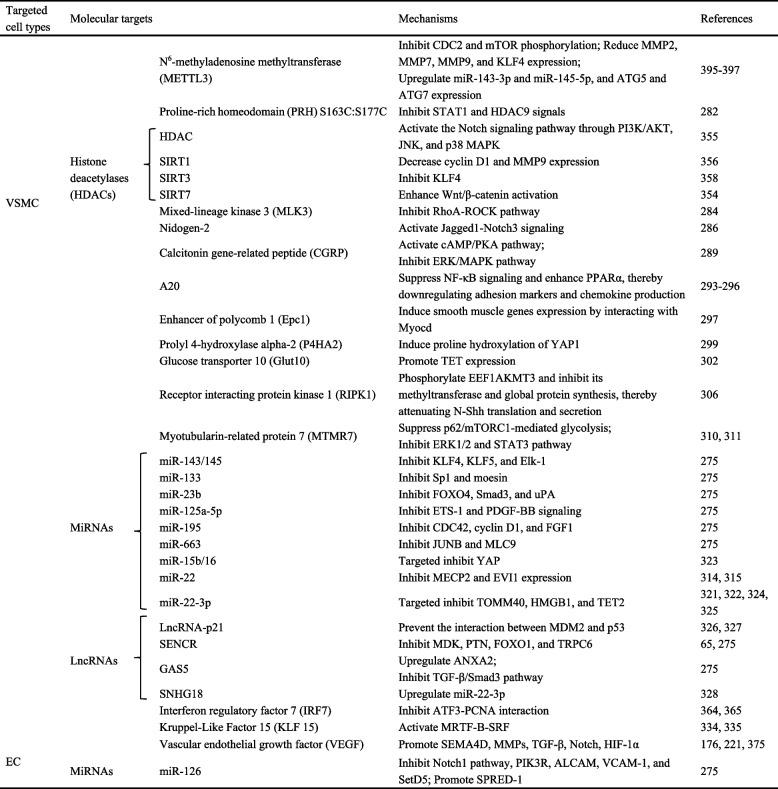
Targets for inhibiting neointima formation and vascular restenosis

### Targeting VSMC phenotypic switching

#### Pro-proliferative and pro-migratory targets in VSMCs

##### Lysine-specific demethylase 1 (LSD1)

LSD1 demethylates mono- and di-methylated histone 3 lysine 4 (H3K4), repressing target gene transcription and playing a key role in cancer [229–232]. LSD1 inhibitors have been developed for cancer treatment [233–236]. Significantly, LSD1 expression is elevated in human and mouse stenotic arteries and in PDGF-BB-treated VSMCs. Knockdown of LSD1 inhibits VSMC proliferation by upregulating the CDK inhibitor p21 [237]. Therefore, LSD1 promotes VSMC proliferation, migration, and post-injury neointima formation by repressing p21. Targeting LSD1 to restore p21 levels presents a promising therapeutic strategy for controlling neointima formation and preventing vascular restenosis.

##### Adenosine kinase (ADK)

ADK phosphorylates adenosine to adenosine monophosphate (AMP), a crucial step in adenosine metabolism, and is implicated in neuroinflammation and liver inflammation [238, 239]. ADK also contributes to cardiovascular diseases [240, 241]; its inhibitor ABT702 protects against CaCl_2_-induced aortic inflammation and abdominal aortic aneurysm formation in mice [242]. Crucially, ADK promotes neointima formation after vascular injury by inducing abnormal DNA hypermethylation to upregulate KLF4 expression, thereby driving VSMC proliferation [243]. Inhibiting ADK to correct epigenetic dysregulation and reduce VSMC proliferation/migration represents a potential therapeutic strategy against vascular restenosis and intimal hyperplasia [244].

##### Phosphoglycerate kinase 1 (PGK1)

PGK1, an essential glycolytic enzyme, also regulates angiogenesis, autophagy, and DNA repair, and is significant in cancer development [245–248]. Recently, it has been reported that PGK1 plays a crucial role in the pathological process of neointima formation after balloon angioplasty. In restenosis models both in vivo and in vitro, PDGF-BB induces the expression of PGK1 through its receptor and the downstream PI3K/AKT pathway, enhancing glycolysis, affecting VSMC behavior, and thus promoting VSMC trans-differentiation and neointima formation [249]. Recent studies indicate that inhibition of PGK1 may significantly reduce neointimal thickness, potentially through suppressing the expression and activation of TGFβ1 and its associated signaling pathways [250]. Inhibiting PGK1 could disrupt metabolic support for neointima formation, reducing VSMC proliferation and alleviating restenosis, making it a promising therapeutic target.

##### Ubiquitin-specific peptidase (USP)

Protein turnover depends on the ubiquitin–proteasome system (UPS) involved in the protein degradation. USPs are major members of the de-ubiquitinating enzyme family. USP10 has a dual role as an oncogene and a tumor suppressor in various human cancers [251, 252]. USP10 promotes the proliferation and migration of lung cancer cells by stabilizing HDAC7 [253]. In vascular injury, USP10 exacerbates neointima formation primarily by stabilizing S-phase kinase-associated protein 2 (Skp2) in VSMCs. Skp2 is the F-box component of the SCF^Skp2^ ubiquitin ligase and targets the CDK inhibitor p27 for poly-ubiquitination and degradation. During neointima formation, USP10-mediated Skp2 stabilization leads to p27 degradation, promoting the cell cycle progression and the proliferation of VSMCs [254]. Inhibiting USP10 disrupts the Skp2-p27 axis, potentially reducing the VSMC proliferation and intimal hyperplasia. The role of USP10 in stabilizing Skp2 demonstrates its potential as a therapeutic target for controlling neointima formation.

USP14 is also implicated in cardiovascular diseases and involved in regulating HA-VSMC phenotypic transformation. USP14 knockout promotes the expression of VSMC contractile markers such as α-SMA, calponin, and smooth muscle myosin heavy chain (SM-MHC) by blocking the mTOR/70kDa ribosomal protein S6 kinase (P70S6K) signaling pathway, hindering the switch to a synthetic phenotype [255]. Recently, USP14 was shown to stabilize Skp2 by reducing its ubiquitination and degradation, thereby promoting HA-VSMCs proliferation, migration, and neointimal hyperplasia. The deficiency of USP14 reduces intimal thickening in mice carotid ligation models, highlighting its crucial role in the pathology of vascular remodeling and its potential as a therapeutic target for arterial restenosis [256].

##### FGF10

**Fig. 1:**
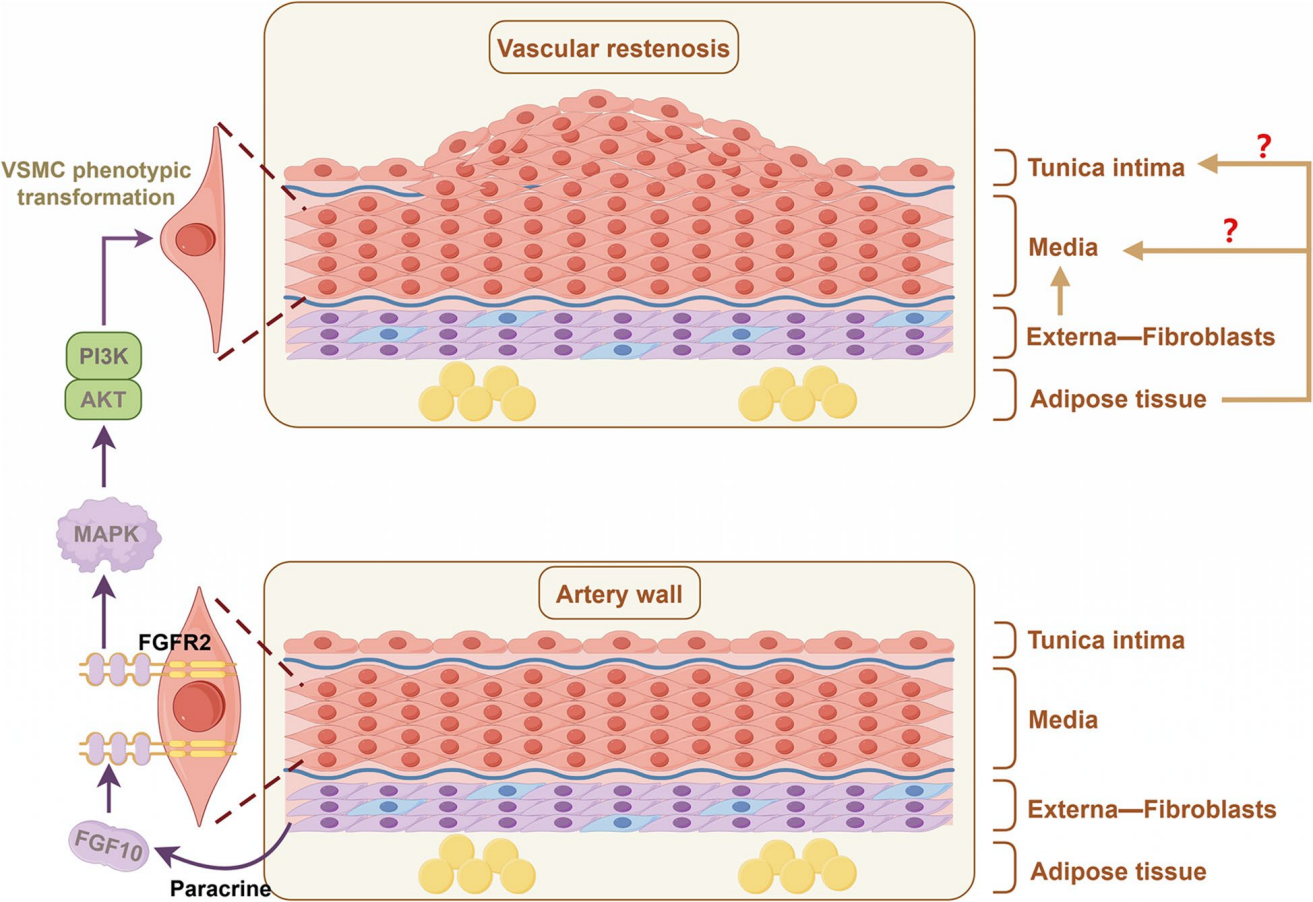
VAFs regulate the phenotypic transformation of VSMCs through paracrine secretion of FGF10. Specifically, FGF10 released by VAFs acts in a paracrine manner on the vascular media, where it binds to FGFR2 on VSMCs. This binding activates the MAPK/PI3K-AKT pathway, promoting VSMC proliferation and migration, and thereby contributing to vascular wall thickening and neointima formation. Although perivascular adipose tissue is known to secrete various bioactive molecules that modulate vascular function, its indirect mechanisms of regulating the medial and intimal layers—and thus influencing vascular restenosis—remain poorly understood. Created in Figdraw.com

FGF10 plays a pivotal role in regulating the behavior patterns of VSMCs and neointima formation [257]. It promotes the proliferation and migration of VSMCs in SHRs via the Wnt/β-catenin pathway [258]. Crucially, vascular adventitial fibroblasts (VAFs), as a major source of FGF10, are located in the outermost layer of the vascular wall and are key participants in vascular remodeling, especially in response to injury. Activated VAFs release FGF10, which acts in a paracrine manner on VSMCs. Binding to its specific receptor FGF receptor 2 (FGFR2), FGF10 activates the MAPK/PI3K-AKT pathway, driving VSMC proliferation, migration, vascular wall thickening, and neointima formation [259] (Fig. 1). As a key regulator of vascular hyperplasia, FGF10 is a promising therapeutic target for vascular restenosis.

##### Telomeric silencing 1-like (DOT1L)

DOT1L is a crucial epigenetic modifier that specifically methylates histone H3 at lysine 79 [260, 261]. This modification plays a crucial role in regulating the complex interactions between VSMCs and monocytes. During VSMC phenotypic transformation, DOT1L upregulation directly promotes the transcription of NF-κB1 and NF-κB2, inducing the expression of inflammatory factors C–C motif chemokine ligand 5 (CCL5) and C-X-C motif chemokine ligand 10 (CXCL10). DOT1L knockout balances the NF-κB pathway by enhancing anti-inflammatory gene expression and suppressing pro-inflammatory genes, thereby effectively curbing excessive inflammation and abnormal VSMC proliferation. This protective mechanism mitigates atherosclerosis and neointima formation [262]. Recently, it has been reported that the DOT1L-targeting inhibitor pinometostat reduces intimal thickening by 76.8% [263]. Although DOT1L-selective inhibitors have been tested in cancer therapy, their application in vascular restenosis still requires further validation, particularly regarding toxicity and safety.

##### Unc-51-like kinase 1 (ULK1)

The expression of ULK1 in HA-VSMCs activates autophagy, promoting cell migration and neointima formation. ULK1 induces autophagic degradation of K-acetyltransferase 2 A (KAT2A), reducing tubulin acetylation and destabilizing the microtubule cytoskeleton. This facilitates directional migration of VSMCs and neointimal hyperplasia [264]. Similar ULK1-mediated autophagy and phenotypic transformation occur in rat VSMCs [265].

##### A disintegrin and metalloprotease 22 (ADAM22)

ADAM22 is a multifunctional transmembrane protein with roles in physiology and diseases [266, 267]. Its association with vascular pathologies is poorly characterized. Current evidence indicates that ADAM22 accelerates VSMCs phenotypic transformation and neointima formation by promoting the phosphorylation of ERK. Inhibiting ADAM22 may thus be a therapeutic strategy against neointimal hyperplasia [268].

##### Sex-determining region Y (SRY)-related HMG-box gene 10 (Sox10)

Sox10 regulates vascular inflammation by activating macrophage-like VSMC transdifferentiation, which contributes to vascular remodeling. The PI3K/AKT signaling pathway mediates the activation of Sox10. G protein signaling 5 (RGS5) blocks the PI3K/AKT/Sox10 phosphorylation cascade by interacting with AKT, thereby suppressing macrophage-like transdifferentiation of VSMCs and reducing vascular inflammation [269].

##### Phospholipase C-β (PLC-β)

Phospholipase C (PLC) is a key effector enzyme in growth factor and vasoconstriction signaling, with 13 isoforms performing distinct cellular functions [270]. At present, PLC-β uniquely regulates VSMC phenotypic transformation. It mediates sphingosine-1-phosphate (S-1-P)-induced ROS generation and VSMC migration by activating MAPK [271]. Recently, it has been reported that PLC-β3 also links Ang II signaling to ROS production via NADPH oxidase 1 (NOX1) activation. PLC-β3-dependent calcium mobilization is essential for NOX/ROS activation, driving Ang II-mediated VSMC proliferation, migration, and vascular restenosis [272].

##### Glucose-6-phosphate dehydrogenase (G6PD)

G6PD, the rate-limiting enzyme of the pentose phosphate pathway (PPP), plays an important role in maintaining the survival of VSMCs [273]. It competes with BCL2-associated X (Bax) for binding to voltage-dependent anion channel 1 (VDAC1), reducing the oligomerization of VDAC1 and counteracting VDAC1-Bax-mediated apoptosis. This mechanism promotes VSMC survival and accelerates vascular neointimal hyperplasia [274].

##### miRNAs

**Fig. 2:**
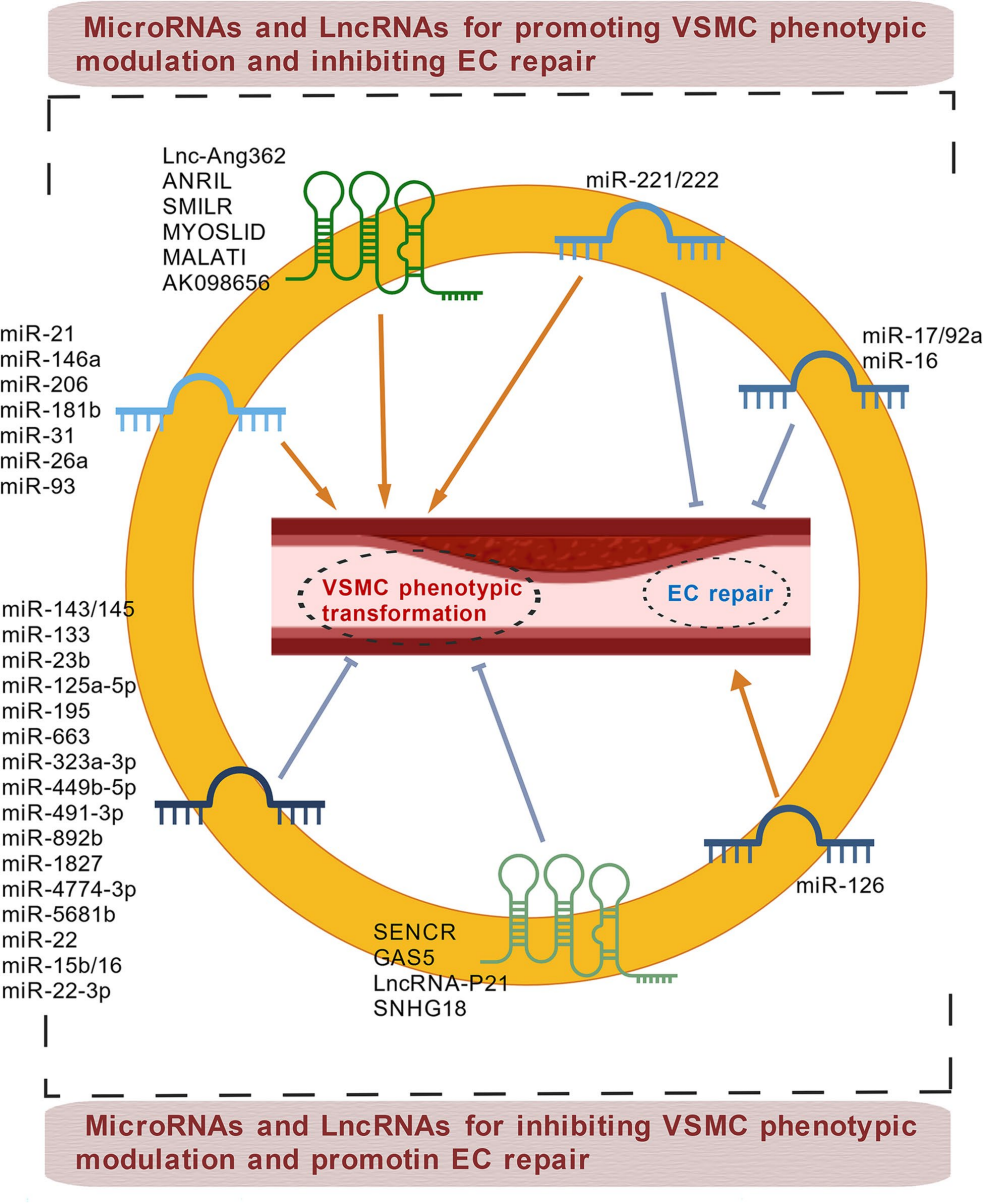
MiRNAs and LncRNAs play dual roles in either promoting or inhibiting vascular restenosis. Among the miRNAs that facilitate VSMC phenotypic switching and contribute to vascular restenosis are miR-21, miR-221/222, miR-146a, miR-206, miR-181b, miR-31, miR-26a, and miR-93. Notably, miR-221/222—along with miR-17/92a and miR-16— also impair endothelial repair and promote vascular intimal thickening. Similarly, several LncRNAs that exacerbate VSMC phenotypic switching and vascular restenosis have been identified, including Lnc-Ang362, ANRIL, SMILR, MYOSLID, MALAT1, and AK098656. On the other hand, certain miRNAs act to suppress VSMC phenotypic switching and inhibit vascular restenosis. These include miR-143/145, miR-133, miR-23b, miR-125a-5p, miR-195, miR-663, miR-323a-3p, miR-449b-5p, miR-491-3p, miR-892b, miR-1827, miR-4774-3p, miR-5681p, miR-22, miR-15b/16, and miR-22-3p. Additionally, miR-126 supports endothelial repair and thereby helps prevent vascular restenosis. Similarly, some LncRNAs such as SENCR, GAS5, LncRNA-p21, and SNHG18 also inhibit VSMC phenotypic switching and reduce restenosis. Created in BioGDP.com

miRNAs are small non-coding RNAs that play crucial roles in regulating gene expression and are increasingly studied in vascular biology. Ciro Indolf et al*.* summarized the role of non-coding RNAs in vascular intimal thickening [275], highlighting key miRNAs that promote VSMC phenotypic transformation and restenosis: miR-21, miR-221/222, miR-146a, miR-206, miR-181b, miR-31, miR-26a (Table 1). Additionally, miR-93 targets mitofusin 2 (Mfn2) through directly binding to the 3' UTR of Mfn2 mRNA, inhibiting its translation and disrupting mitochondrial function. This elevates ROS production, induces a pro-proliferative metabolic shift, and drives neointima formation [276]. Clinically, plasma miR-93-5p levels are elevated in atherosclerosis patients with ISR, suggesting its utility as a potential ISR biomarker [277]. Therefore, miRNA targeting holds promise for treating vascular restenosis (Fig. 2).

##### LncRNAs

Studies [65, 275] also summarized that LncRNAs, such as Lnc-Ang362, ANRIL, SMILR, MYOSLID, MALAT1, and AK098656, promote VSMC phenotypic transformation (Table 1). SMILR enhances the proliferation and migration of VSMCs by suppressing miR-141 and activating the RhoA/ROCK signaling pathway [278]. MALAT1 drives the proliferation of VSMCs by targeting the miR125a-5p/STAT3 axis [279] (Fig. 2). Beyond their direct effects on gene expression, many of these pro‑restenotic LncRNAs function as competing endogenous RNAs (ceRNAs) or molecular sponges for anti‑proliferative miRNAs, thereby derepressing miRNA targets and further driving VSMC phenotypic switching. For instance, MALAT1, as mentioned above, not only acts through the miR‑125a‑5p/STAT3 axis but also serves as a ceRNA for other miRNAs such as miR‑145‑5p, whose inhibition promotes VSMC proliferation [280]. Similarly, ANRIL has been reported to sponge miR-449a, a key regulator of the cell cycle and cell proliferation, thereby activating CDK6 and accelerating cell proliferation [281]. These findings illustrate a hierarchical ceRNA network: LncRNAs can sequester protective miRNAs, leading to the derepression of pro‑proliferative targets. Therefore, targeting the interaction between these LncRNAs and their cognate miRNAs (e.g., using antisense oligonucleotides to block the sponge effect) represents a novel therapeutic strategy to restore the activity of endogenous anti‑restenotic miRNAs and simultaneously fine‑tune multiple downstream pathways.

**Fig. 3:**
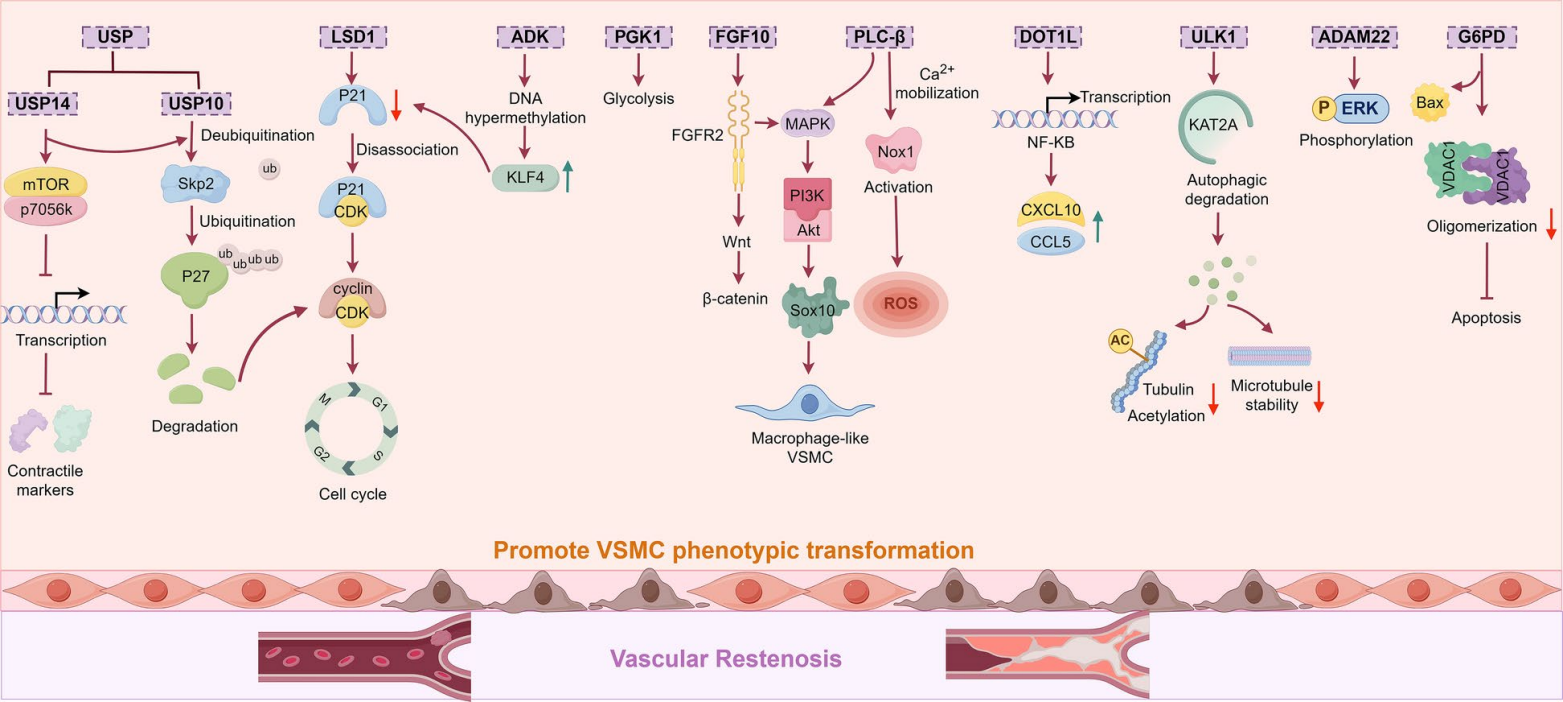
Regulatory network of protein molecules targeting the promotion of VSMC phenotypic switching and vascular restenosis. LSD1, ADK, and USP10 collectively regulate p21/p27 to drive cell cycle progression and proliferation. USP14 inhibits the VSMC contractile phenotype via the mTOR pathway. FGF10 and PLC-β cooperate to promote VSMC transdifferentiation toward a macrophage-like state through the MAPK pathway, while also enhancing VSMC proliferation and migration via the Wnt and Nox1 pathways, respectively. DOT1L and G6PD regulate VSMC inflammation and apoptosis by promoting chemokine expression and reducing mitochondrial VDAC1 oligomerization, respectively. ULK1 modulates cytoskeletal stability through KAT2A-mediated autophagic degradation, thereby facilitating cell migration. PGK1 and ADAM22 further contribute to VSMC phenotypic switching by regulating glycolysis and ERK signaling, respectively. Created in Figdraw.com

The aforementioned targets collectively drive the phenotypic switch of VSMCs from a contractile to a synthetic state—ultimately leading to vascular restenosis—by interfering with diverse molecular mechanisms, including VSMC cell cycle regulation (LSD1, ADK, USP), metabolism (PGK1, G6PD), inflammation (DOT1L), protein stability (ULK1, USP), and signal transduction (FGF10, PLC-β, ADAM22) (Fig. 3). Among these targets, some have advanced to the preclinical stage, demonstrate considerable therapeutic potential, and already possess well-characterized inhibitors, such as those targeting LSD1, ADK, and DOT1L. Others remain at the basic research stage, facing challenges including the presence of multiple isoforms, limited specificity, and potential cytotoxic effects, which hinder their progression toward preclinical evaluation. Additionally, certain targets (e.g., SOX10, G6PD) exhibit low drugability and higher safety risks, rendering them less feasible for clinical translation (Table 3). Furthermore, this review highlights several miRNAs and LncRNAs that significantly promote vascular restenosis, indicating promising potential for drug development targeting these molecules. For instance, miR-93-5p serves dual roles as both a predictive biomarker for restenosis and a potential therapeutic target.

**Table 3:** Comparative features of targets that promote or inhibit VSMC phenotypic switching

Targets	Advantages	Disadvantages	Research Stage
Pro‑restenotic targets			
LSD1	LSD1 inhibitors have entered clinical trials and do not possess direct cytotoxicity	Potential cardiotoxicity and off-target risks	Pre-clinical exploration; Candidate target
ADK	Commercially available inhibitors exist; Enhances endogenous adenosine system	Adenosine receptors are widespread, leading to potential broad side effects; Lacks receptor selectivity	Pre-clinical exploration; Candidate target
PGK1	Commercially available inhibitors exist; PGK1 inhibitors exert anti-proliferative effects through multiple pathways and improve the cellular microenvironment	Risk of multi-tissue toxicity; May trigger compensatory pathway activation	Basic research stage; Innovative research target
USP	USP inhibitors have entered clinical trials, and have potential to overcome drug resistance	Multiple subtypes with complex functions; Potential cytotoxicity	Basic research stage; Innovative research target
FGF10	FGF receptor inhibitors have entered clinical trials; Targeting FGF10 is relatively specific	Currently no FGF10-specific inhibitors exist; Risk of drug resistance	Basic research stage; Innovative research target
DOT1L	DOT1L inhibitors have entered clinical trials; Its structure is favorable for drug design	Potential cytotoxicity; Risk of drug resistance	Pre-clinical exploration; Candidate target
ULK1	Commercially available inhibitors exist; Integrates multiple upstream signals, making it a relatively efficient target	Autophagy is a double-edged sword, requiring extremely precise regulation	Basic research stage; Innovative research target
ADAM22	A scaffold protein; Upstream in multiple signaling pathways	Few related reports; Difficult to develop inhibitors	Basic research stage; Innovative research target
SOX10	A transcription factor; Directly regulates VSMC phenotype	Low drugability; High safety risks associated with inhibition	Limited supporting data; Low feasibility
PLC-β	Commercially available inhibitors exist; Good drugability; Well-defined mechanism	Different subtypes with functional overlap; Potential systemic side effects	Basic research stage; Innovative research target
G6PD	Commercially available inhibitors exist; Good drugability; Highly expressed in abnormally proliferating cells	Contraindicated in patients with G6PD deficiency; Potential systemic side effects	Limited supporting data; Low feasibility
PERK	Commercially available inhibitors exist; Good drugability; Multiple effects	Affect ER stress; Potential safety risks associated with inhibition	Basic research stage; Innovative research target
Protective targets for the VSMC contractile phenotype			
PRH S163C:S177C	A transcription factor regulating multiple downstream signaling pathways	Potential off-target and cytotoxic risks; Low drugability	Limited supporting data; Low feasibility
MLK3	Regulates multiple pathological processes with a clear mechanism	Belongs to a protein family with functional redundancy and compensatory risks	Basic research stage; Innovative research target
Nidogen-2	Targets cell-matrix interactions; Regulate proliferation and migration	Poor drugability; May interfere with normal cellular repair functions	Basic research stage; Innovative research target
CGRP	Clinically available CGRP receptor antagonists/ antibodies; Good drugability; Exerts protective effects through multiple pathways	Risk of systemic side effects	Pre-clinical exploration; Candidate target
A20	An endogenous negative feedback regulator with multi-faceted protective effects	Poor drugability; Potential off-target risk	Pre-clinical exploration; Candidate target
Epc1	Involved in epigenetic regulation, enabling long-term effects	Complex functionality; Off-target and carcinogenic risks; Poor drugability	Limited supporting data; Low feasibility
P4HA2	Targets hydroxylation post-translational modification with relatively low cytotoxicity; Commercially available inhibitors; Good drugability	Multiple family members; Compensatory risks	Basic research stage; Innovative research target
Glut10	Regulates intracellular VitC balance, maintaining antioxidant defenses	Poor drugability; Risks associated with overactivation	Basic research stage; Innovative research target
RIPK1	Commercially available inhibitors; Good drugability; Targets cell fate regulation	Potential systemic side effects	Basic research stage; Innovative research target
MTMR7	Regulates cellular homeostasis by targeting lipid metabolism and dephosphorylation reactions	Multiple family members with functional redundancy and compensatory risks; Moderate drugability	Basic research stage; Innovative research target

#### Anti-proliferative and anti-migratory targets in VSMCs

##### Proline-rich homeodomain (PRH) S163C:S177C

PRH S163C:S177C, also known as hematopoietically expressed homeobox (HHEX), is a highly conserved transcription factor crucial for cell development and differentiation, acting via transcriptional and post-transcriptional mechanisms. It has been reported that PRH S163C:S177C attenuates the proliferation and migration of human saphenous vein VSMCs through the STAT1 and HDAC9 signaling pathways, while promoting endothelial repair and inhibiting inflammation [282].

##### Mixed-lineage kinase 3 (MLK3)

MLK3, a key member of the MAPK kinase family [283], plays a crucial role in regulating VSMC function and serves as a vital inhibitor of neointima formation. MLK3 precisely regulates the signaling pathway of the small GTPase RhoA, maintaining the proliferation and migration of VSMCs within normal ranges to prevent excessive intimal hyperplasia. However, MLK3 deficiency leads to abnormal RhoA pathway activation, triggering hyperactive VSMC proliferation and migration, thereby accelerating neointima formation. In addition, MLK3 deficiency may indirectly impair EC function, further exacerbating VSMCs abnormalities and promoting neointima formation [284]. Therefore, MLK3 represents a promising target for inhibiting neointima, potentially offering novel therapeutic approaches for vascular remodeling-related diseases with significant clinical value.

##### Nidogen-2

Nidogen-2 is a crucial vascular protective factor that inhibits neointima formation after vascular injury through multiple mechanisms [285]. As a matrix protein, Nidogen-2 not only maintains the contractile phenotype of VSMCs by interacting with extracellular matrix components, thereby limiting cell proliferation and migration, but also acts as a bridging molecule between the Jagged1 and Notch3 signaling pathways, enhancing the activation of Notch3 signaling. This process further consolidates the contractile phenotype of VSMCs and inhibits the switch to a synthetic phenotype. In vascular injury models, Nidogen-2 overexpression significantly inhibited neointima formation, while its deficiency promoted VSMC phenotypic transformation, proliferation, and migration, and exacerbated neointima thickening [286]. These findings highlight the importance of Nidogen-2 as a target for inhibiting vascular neointima, providing a scientific basis and potential therapeutic strategy for maintaining vascular stability and preventing restenosis.

##### Calcitonin gene-related peptide (CGRP)

As a vasculoprotective molecule, CGRP represents a promising therapeutic target for cardiovascular diseases [287, 288]. CGRP potently inhibits neointimal hyperplasia following vascular injury through a dual pathway mechanism. Specifically, it effectively reduces oxidative stress induced by vascular injury and decreases ROS levels by activating the cAMP/PKA signaling pathway. This protects vascular ECs and VSMCs from damage, preserving their normal function. Supporting this, CGRP knockout mice exhibit significantly enhanced neointima formation following vascular injury, alongside elevated oxidative stress markers, including decreased eNOS expression, increased p47phox expression, elevated 4HNE levels, and enhanced macrophage infiltration [289]. Furthermore, CGRP directly inhibits the phenotypic switch of VSMCs from contractile to synthetic by increasing intracellular cAMP and activating PKA. It also downregulates the expression of cell cycle-related proteins such as Cyclin D1 and inhibits the activation of pro-proliferative signaling pathways such as ERK/MAPK, thus limiting VSMC proliferation and migration. This mechanism effectively prevents excessive proliferation of neointima and vascular restenosis [289]. Recent studies show that co-culturing rat bone marrow mesenchymal stem cells (MSCs) overexpressing CGRP with VSMCs significantly inhibits the proliferation and migration of VSMCs. This finding supports the potential application of CGRP gene-engineered MSCs in the treatment of restenosis [290].

##### A20

A20 (also known as TNFAIP3), a key negative regulator of the NF-κB pathway, exerts protective effects in various diseases by inhibiting inflammatory signaling and significantly suppresses vascular neointima formation [291, 292]. This homeostatic protein downregulates adhesion markers and chemokines required for macrophage migration to vascular injury sites and inhibits adventitial angiogenesis, thereby reducing inflammatory macrophage infiltration and preventing neointimal hyperplasia [293]. Additionally, A20 inhibits the activation of the NF-κB signaling pathway via its deubiquitinating activity, effectively regulating inflammatory responses and reducing excessive inflammatory factor production, thus alleviating local inflammation. Beyond suppressing abnormal VSMC proliferation and migration, A20 further regulates inflammation and cell proliferation-related signaling pathways by enhancing the activity of PPARα, successfully suppressing vascular restenosis and validating its potential as a therapeutic target [294]. Recent reports confirm that upregulated A20 expression inhibits excessive proliferation and migration of PASMCs by blocking the NF-κB signaling pathway [295, 296]. In summary, A20 effectively inhibits post-injury neointima formation through multifaceted mechanisms—reducing inflammation, inhibiting macrophage infiltration and VSMC proliferation and migration, and regulating the PPARα signaling pathway—offering novel therapeutic strategies for treating vascular diseases like vascular restenosis.

##### Enhancer of polycomb 1 (Epc1)

Current evidence suggests Epc1 may be a novel negative regulator of neointima formation after carotid artery injury. As an important component of the polycomb complex, Epc1 functions epigenetically. It significantly enhances the transcriptional activation capacity of the potent co-activator myocd through direct binding. This promotes the up-regulation of smooth muscle genes (e.g., α-SMA, SM22α and Cav-1) and accelerates VSMC transition towards a stable contractile phenotype, effectively curbing the hyperplasia of neointima [297]. Epc1 plays a significant role in maintaining the contractile phenotype of VSMCs and represents a novel inhibitory target for treating vascular restenosis.

##### Prolyl 4-hydroxylase alpha-2 (P4HA2)

P4HA2, a key enzyme in protein post-translational modification, is a potential prognostic tumor biomarker and promising therapeutic target [298]. Recent studies have found that P4HA2 restricts the proliferation of VSMCs and neointima formation by inducing proline hydroxylation of YAP1, thus inhibiting atherosclerosis and restenosis. This mechanism represents a promising therapeutic approach for traumatic cardiovascular diseases [299].

##### Glucose transporter 10 (Glut10)

Glut10 contributes to maintain the contractile phenotype of SMCs [300, 301]. Glut10 expression is significantly downregulated in restenotic arteries of humans and mice. Knocking out Glut10 accelerates neointimal hyperplasia, while its overexpressing in the carotid artery attenuates neointima formation. Glut10 promotes the expression and activation of TET, increases the concentration of mitochondrial VitC, induces mitochondrial DNA (mtDNA) demethylation, and improves mitochondrial function, thus preventing the progression of neointimal hyperplasia. Consequently, modulating the Glut10-TET2/3 axis is a promising therapeutic direction for restenosis [302].

##### Receptor interacting protein kinase 1 (RIPK1)

RIPK1 plays a crucial role in inflammation and cell death [303–305]. Recent studies indicate its involvement in controlling vascular restenosis. RIPK1 phosphorylates EEF1A lysine methyltransferase 3 (EEF1AKMT3), inhibiting its methyltransferase activity and global protein synthesis. This weakens the translation and secretion of endothelial N-terminal Sonic Hedgehog (N-Shh), ultimately suppressing SMC proliferation and neointima formation [306]. However, in a heat-mediated rat vascular injury model, elevated RIPK1 and PIPK3 expression induces abnormal VSMC phenotypes, and reducing their expression restores the contractile phenotype. Necrostatin-1 ameliorates necroptosis and maintains the contractile phenotype of VSMCs by inhibiting this RIPK1/PIPK3 overexpression [307]. Thus, RIPK1's role may vary across vascular injury models, and its specific function in regulating VSMC phenotype and vascular restenosis requires further validation in diverse experimental settings.

##### Myotubularin-related protein 7 (MTMR7)

Myotubularins (MTMs), a phosphatase family acting on phosphatidylinositols, are widely expressed in blood vessels and regulate cell proliferation, survival, endocytosis, and autophagy [308, 309]. Studies show that the expression of MTMR7 is significantly down-regulated in injured arteries. Overexpressing MTMR7, both in vivo and in vitro, significantly inhibits glycolysis and the activity of mTORC1 in PDGF-BB-stimulated VSMCs. This leads to the dephosphorylation and dissociation of p62 from mTORC1, ultimately inhibiting VSMCs proliferation and migration and protecting against excessive intima thickening [310]. Recent reports further demonstrate that MTMR7 significantly inhibits the phosphorylation levels of ERK1/2 and STAT3, thereby suppressing PASMCs proliferation and migration [311].

##### miRNAs

The previous section discussed miRNAs that promote neointimal hyperplasia and restenosis. As summarized by previous studies [275, 312], other miRNAs, including miR-143/145, miR-133, miR-23b, miR-125a-5p, miR-195, miR-663, miR-323a-3p, miR-449b-5p, miR-491-3p, miR-892b, miR-1827, miR-4774-3p, and miR-5681b, inhibit VSMC phenotypic transformation and restenosis (Fig. 2). In addition, miR-22 has emerged as a new key regulator in vascular biology, particularly in suppressing VSMC phenotypic switching and neointima formation [313]. It achieves this by precisely targeting and inhibiting two critical molecules, methyl-CpG-binding protein 2 (MECP2) and ecotropic viral integration site 1 (EVI1). Through RNA silencing, miR-22 reduces the protein synthesis of MECP2 and EVI1. This action not only prevents VSMC transition from a stable contractile phenotype to a highly proliferative synthetic phenotype but also impairs their capacity for pathological proliferation and migration. In vascular injury models, elevated miR-22 expression is strongly associated with significantly reduced neointimal thickness, underscoring its pivotal role in suppressing abnormal proliferation and migration of VSMCs and neointimal hyperplasia [314, 315]. This discovery enhances our understanding of the molecular mechanisms underlying vascular restenosis and provides a molecular basis for developing miR-22-based precision therapies targeting this pathology. The shift toward localized delivery systems is necessitated by the inherent limitations of systemic miRNA therapy. Unprotected miRNAs are rapidly degraded by serum RNases, cleared by the reticuloendothelial system, and may trigger off-target effects or immune responses. These barriers drastically reduce bioavailability at the injured vessel wall and increase the risk of systemic toxicity. Thus, stent-based or hydrogel-based local delivery provides a rational strategy to achieve sustained, lesion-specific miRNA activity while circumventing the pitfalls of systemic administration. Recent therapeutic strategies utilize localized delivery systems such as PEI/PAA/miR-22-coated balloons, miR-22-coated stents, or miR-22-loaded laponite hydrogels to treat vascular restenosis [316–318]. These approaches enable the sustained release of miR-22, inhibiting MECP2, reducing VSMCs proliferation, and decreasing intimal hyperplasia following vascular injury, demonstrating miR-22's significant therapeutic potential for cardiovascular diseases. In addition, some compounds also maintain VSMC contractile phenotype and prevent intimal thickening by regulating miR-22. For instance, palmitic acid upregulates miR-22, thereby inhibiting the switch of VSMCs to the synthetic phenotype [319]. Although demonstrated in vitro, this finding highlights miR-22's therapeutic promise [319]. Similarly, curcumin increases the expression of miR-22, directly targeting the SP1 transcription factor in VSMCs to negatively regulate VSMC proliferation and migration, ultimately inhibiting intimal thickening [320].

Clinical data indicate that miR-22-3p expression is downregulated in the peripheral blood of patients with ISR. A negative targeting relationship exists between miR-22-3p and the mitochondrial outer membrane protein TOMM40, leading to elevated TOMM40 levels in ISR patients. This upregulation promotes the proliferation, migration, and phenotypic transformation of VSMCs. Overexpression of miR-22-3p can inhibit VSMC phenotypic transformation by targeting TOMM40, suggesting its potential as a therapeutic candidate for preventing restenosis after PCI in cardiovascular patients [321]. Additionally, miR-22-3p has been reported to inhibit VSMCs proliferation and migration by targeting high mobility group box 1 (HMGB1), thereby playing a critical role in attenuating neointimal hyperplasia [322]. Collectively, these findings demonstrate that miR-22 inhibits VSMC phenotypic transformation through multiple pathways, helping maintain the contractile phenotype of VSMCs and ultimately reducing neointimal thickening.

Emerging evidence indicates that miR-15b/16 acts as a key regulatory factor in suppressing neointima formation following vascular injury. By directly targeting and inhibiting YAP—a core effector of the Hippo signaling pathway—miR-15b/16 effectively counteracts its pro-proliferative and phenotypic switching effects on VSMCs. Additionally, miR-15b/16 promotes the expression of contractile phenotype-related genes, including α-SMA, SM22α, and SM heavy chain myosin, thus effectively attenuating excessive neointima formation after vascular injury. Conversely, inhibiting of miR-15b/16 produces the opposite effect [323]. In addition, the activity of these protective miRNAs is itself tightly regulated by the ceRNA network. In this network, LncRNAs and circular RNAs (circRNAs) act as molecular sponges that sequester miRNAs, thereby derepressing miRNA targets. For example, circular RNA mitogen-activated protein kinase kinase kinase 5 (circMAP3K5) promotes VSMC differentiation by sponging miR-22-3p, thus upregulating its target TET2 [324, 325]. This exemplifies a prototypical ceRNA mechanism.

##### LncRNAs

Whereas the previous section focused on LncRNAs that promote vascular restenosis, several others exert protective effects by inhibiting this process (Fig. 2). As summarized by previous studies, certain LncRNAs—such as SENCR and GAS5— inhibit VSMC phenotypic transformation [65, 275]. Additionally, in the context of atherosclerosis research, LincRNA-p21 has been identified as a key suppressor of neointima formation. Overexpression of LincRNA-p21 significantly reduces neointimal hyperplasia. Mechanistic studies reveal that VSMCs overexpressing LincRNA-p21 exhibit enhanced p53 activity. Specifically, LincRNA-p21 directly binds to the E3 ubiquitin ligase mouse double minute 2 (MDM2), interfering with the MDM2-p53 interaction. This stabilizes p53 by limiting its ubiquitination and degradation, leading to accumulation of p53 protein and subsequent upregulation of its downstream target genes. These include regulators of the cell cycle (e.g., p21), apoptosis (e.g., Bax), and anti- proliferative pathways, collectively inducing cell cycle arrest and apoptosis, thereby attenuating neointimal thickening. Conversely, knockdown of LincRNA-p21 produces the opposite effects, underscoring its essential role in modulating p53 transcriptional activity to limit neointima formation [326, 327]. Another LncRNA, small nucleolar RNA host gene 18 (SNHG18), promotes the contractile VSMC phenotype by upregulating miR-22-3p, suggesting its potential as a novel regulator of the contractile phenotype and a suppressor of injury-induced intimal hyperplasia. Therefore, targeting the SNHG18/miR-22-3p signaling axis may hold therapeutic promise for vascular diseases [328]. Collectively, LncRNAs and circRNAs can control the availability of anti‑proliferative miRNAs, which then govern a network of pro‑contractile target genes. Notably, this ceRNA logic operates in both directions. In contrast to the pro‑restenotic ceRNA mechanisms described in Section "Pro-proliferative and pro-migratory targets in VSMCs" (where LncRNAs such as MALAT1 and ANRIL sponge protective miRNAs to promote VSMC proliferation), the examples here highlight how certain LncRNAs and circRNAs (e.g., circMAP3K5) can actively protect the contractile phenotype by sponging and preserving the levels of anti‑restenotic miRNAs. Therefore, future therapeutic strategies aimed at modulating these ceRNA interactions—for instance, disrupting a pro‑restenotic LncRNA‑miRNA interaction, or overexpressing a protective LncRNA or circRNA—hold promise for restoring the balance of miRNA networks and simultaneously fine‑tuning multiple downstream pathways to prevent vascular restenosis.

**Fig. 4:**
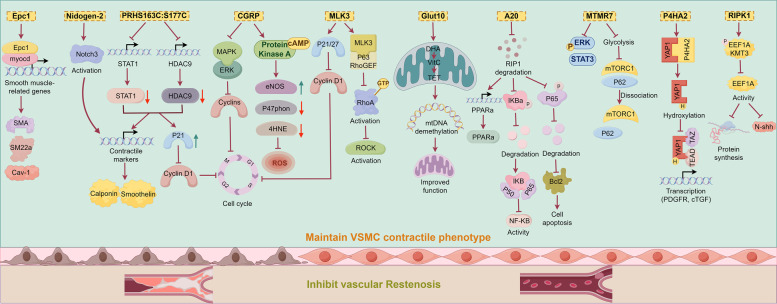
Regulatory network of protein molecules targeting the reduction of VSMC phenotypic switching and vascular restenosis. CGRP, PRH S163C:S177C, and MLK3 cooperatively regulate p21/p27 or cyclins to inhibit cell cycle progression and maintain the VSMC contractile phenotype. Epc1, Nidogen-2, and PRH S163C:S177C promote the expression of VSMC contractile markers through distinct signaling pathways. CGRP additionally alleviates VSMC oxidative stress via PKA signaling. MLK3 also impedes VSMC cytoskeletal dynamics and migration by inhibiting the Rho-ROCK pathway. Glut10 helps preserve the contractile phenotype by protecting mitochondrial function. MTMR7 regulates the VSMC contractile phenotype through both ERK and mTOR pathways. P4HA2 and RIPK1 inhibit VSMC phenotypic switching by reducing protein synthesis and secretion. Finally, A20 exerts multi-faceted inhibitory effects by suppressing NF-κB-mediated inflammation and Bcl2 signaling, while simultaneously promoting PPAR signaling. Created in Figdraw.com

The targets described in 4.1.2 collectively maintain the contractile phenotype of VSMCs and suppress their abnormal proliferation and migration through diverse molecular mechanisms. These include inhibiting cell cycle progression (PRH S163C:S177C, CGRP, MLK3), promoting the contractile phenotype (Epc1, Nidogen-2), suppressing inflammation (A20), improving mitochondrial function (Glut10), and reducing secretory activity (RIPK1, P4HA2). Together, they form a multi‑layered regulatory network that effectively counteracts neointimal hyperplasia and restenosis following vascular injury (Fig. 4). Among these, the targets CGRP and A20 have entered the preclinical stage. CGRP exhibits favorable drugability, with corresponding antibodies and antagonists already available for clinical use (Table 3). Although A20 itself shows limited drugability, it acts as an endogenous negative‑feedback inhibitor with considerable protective potential. Targets such as MLK3, Nidogen‑2, P4HA2, Glut10, RIPK1, and MTMR7 remain at the basic research stage. Among them, MLK3, P4HA2, and MTMR7 belong to protein families with multiple members, resulting in low specificity and potential compensatory risks. Nidogen‑2 and Glut10 face challenges in drugability (Table 3). RIPK1, while considered druggable, exhibits controversial roles across different vascular injury models [306, 307]. Finally, the protective roles of PRH S163C:S177C and Epc1 are currently supported by limited experimental data, rendering their clinical translation less likely at this stage (Table 3).

In contrast to protein-based targets, non-coding RNAs (particularly miRNAs) form a delicate, multi-target regulatory network by leveraging their inherent post‑transcriptional and epigenetic regulatory capabilities, demonstrating unique value in suppressing neointimal hyperplasia. For example, miR‑22 coordinately inhibits the phenotypic switching of VSMCs by simultaneously targeting multiple key molecules such as MECP2, EVI1, TOMM40, and HMGB1. Targeting these non‑coding RNAs offers a highly promising and precise therapeutic strategy for the effective treatment of vascular restenosis.

#### Dual-action targets in VSMCs

##### KLFs

**Fig. 5:**
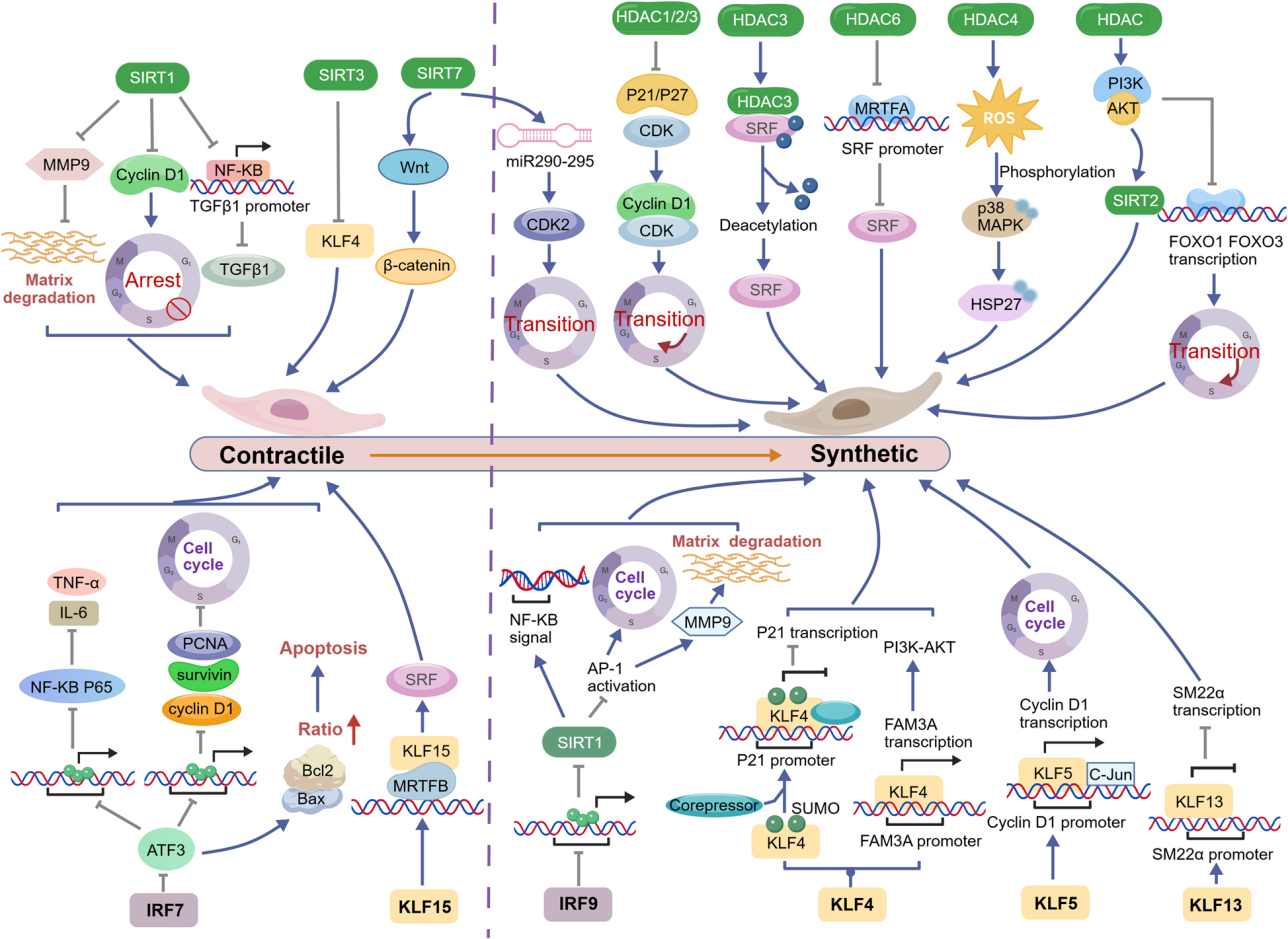
Dual-action molecules (KLFs, HDACs, and IRFs) regulates VSMC phenotypic switching in an isoform-specific manner. Pro‑proliferative HDAC1/2/3 drive G1/S progression by repressing p21/p27 and activating cyclin D1; HDAC3 further promotes proliferation via facilitating SRF deacetylation; HDAC6 reduces SRF transcription by inhibiting MRTF-A, which promotes VSMC phenotypic switching; HDAC4 activates the p38MAPK/HSP27 pathway through enhancing ROS production; and HDACs upregulate SIRT2 via PI3K/AKT while repressing FOXO1 and FOXO3 transcription. Conversely, SIRT1 suppresses NF‑κB/TGF‑β1, cyclin D1, and MMP9 to inhibit VSMC proliferation and migration. SIRT7 exhibits dual roles: Anti‑proliferative via Wnt/β‑catenin but pro‑proliferative via miR290‑295/CDK2. Among KLFs, KLF4, KLF5, and KLF13 promote VSMC synthetic phenotype. Specifically, KLF4 recruits transcriptional corepressors to the P21 promoter via SUMOylation, thereby inhibiting P21 transcription and expression. Alternatively, it directly binds to the FAM3A promoter to enhance FAM3A transcription, which in turn activates the PI3K/AKT pathway. KLF5 forms a complex with c-Jun to activate the cyclin D1 promoter, promoting cyclin D1 transcription and leading to excessive VSMC proliferation. KLF13 directly binds to the SM22α promoter, inhibits SM22α expression, and mediates VSMC dedifferentiation. Whereas KLF15 (via MRTF‑B/SRF) maintains the contractile phenotype. IRF7 suppresses inflammatory and cell cycle signaling by inhibiting the transcription factor ATF3, and promotes VSMC apoptosis by upregulating the Bcl2/Bax ratio, thereby maintaining the contractile phenotype. In contrast, the IRF9 isoform exerts opposing effects: It promotes inflammation, cell cycle progression, and extracellular matrix degradation by suppressing SIRT1. Created in BioGDP.com

KLFs are zinc-finger transcription factors regulating gene activation and repression. Specific members (KLF4, KLF5, KLF13, and KLF15) drive VSMC phenotypic transformation and intimal hyperplasia [329]. Studies have found that SUMOylated KLF4 recruits transcriptional co-repressors to the p21 promoter, inhibiting p21 transcription and expression, thus promoting VSMC proliferation and phenotypic switching [330]. KLF4 also directly binds to the promoter of the metabolic cytokine FAM3A (Family with sequence similarity 3, member A), promoting its transcription and activating the PI3K/AKT pathway, thereby inducing VSMC proliferation and migration [331]. KLF5, in complex with c-Jun, activates the promoter activity of cyclin D1, promoting cyclin D1 transcription and excessive VSMC proliferation [332]. KLF13 directly binds to the promoter of SM22α, repressing its expression and mediating PDGF-BB-induced VSMC dedifferentiation, which plays an important role in the development of atherosclerosis and post-injury restenosis [333]. The large KLF family exhibits functional diversity; in contrast to the pro-restenotic roles of KLF4, KLF5, and KLF13, other members such as KLF15 may have opposite effects (Fig. 5). KLF15 is widely expressed in VSMCs of both arteries and veins and acts as a key inhibitor of VSMC proliferation and migration within the neointima, thereby helping to suppress excessive vascular remodeling [334]. Recent studies further demonstrate that KLF15 interacts with the myocd-related transcription factor B (MRTF-B) to activate SRF, thereby driving the expression of contractile genes in VSMCs and helping maintain their contractile phenotype [335]. These mechanisms underline the therapeutic potential of KLF15 in preventing vascular restenosis. This heterogeneity complicates drug development, as achieving subtype-specific targeting is challenging, making KLF less specific or preferred targets for neointimal hyperplasia and restenosis.

##### HDACs

HDACs regulate gene expression, chromatin remodeling, and cell cycle progression. By deacetylating histones and other proteins, they repress transcription and contribute to cancer [336]. HDAC inhibitors are established cancer therapeutics [337]. Their role in vascular homeostasis and diseases, particularly in regulating ECs and VSMCs, is increasingly recognized, and HDAC inhibitors show efficacy in the treatment of vascular diseases [338]. However, it's important to note that while HDAC inhibitors impede EC proliferation and migration and angiogenesis (reducing tumor growth), this effect hinders re-endothelialization in vascular restenosis [339–341].

In VSMCs, HDAC significantly influence phenotype (Fig. 5). HDAC7 mediates mechanically-induced VSMC migration, inhibitable by HDAC inhibitors [342]. HDAC4 promotes PDGF-BB-induced VSMC proliferation and migration through activating the p38 MAPK/heat shock protein 27 (HSP27) signaling pathway, contributing to neointimal hyperplasia [343]. The study by Zhang et al*.* showed that HDAC6 inhibition promotes VSMC differentiation in vitro through stimulating the MRTF-A/SRF pathway, reducing neointima in injured carotid arteries [344]. HDAC3 directly interacts with SRF, deacetylating it to promote VSMC proliferation and neointima formation, and also regulates endothelial-mesenchymal transition in intimal hyperplasia [198, 345].

In addition, studies have confirmed that HDAC has a significant impact on the cell cycle (Fig. 5). HDAC1, 2, and 3 promote the mitosis of VSMCs and facilitate G1/S transition by regulating CDK inhibitors p21 and p27, as well as promoting the expression of cyclin D1. HDAC inhibitors counteract this: Scriptaid arrests VSMCs in G1 phase, inhibiting proliferation (though its toxicity limits clinical use for restenosis) [201, 346]; Butyrate promotes G1 phase arrest by inhibiting PI3K/AKT pathway and activating forkhead box O1 (FOXO1) and forkhead box O3 (FOXO3) transcription [347, 348]; Apicidin induces G1 arrest by inhibiting the activities of class I/II HDACs [349]; The class I-selective inhibitor MGCD0103 suppresses VSMC proliferation and reduces pulmonary artery pressure in rats [350]. Thus, inducing G1 arrest by targeting cell cycle regulators like cyclin D1 is a primary mechanism by which HDAC inhibitors prevent VSMC proliferation.

Sirtuins (SIRTs), class III HDACs (SIRT1 to SIRT7), regulate cellular senescence, metabolism, oxidative stress, and cancer [351]. SIRT2 and SIRT7 have been reported to be linked to vascular restenosis and neointimal thickening. Increased SIRT2 promotes VSMC proliferation and phenotypic modulation, while its knockdown inhibits this [352]. In addition, SIRT7 deficiency attenuates the proliferation of VSMCs by regulating the miRNA 290–295/CDK2 axis, thereby inhibiting post-injury neointima formation [353]. However, conflicting reports exist regarding SIRT7's role, with some suggesting it inhibits VSMC proliferation and migration via the Wnt/β-catenin-dependent pathway [354]. This discrepancy may arise from differences in experimental models.

The role of HDACs in vascular restenosis is controversial. Although they are often considered promoters of restenosis, and inhibitors are thus proposed as therapeutic agents, other studies present opposing evidence. These studies indicate that HDACs can inhibit the proliferation and migration of VSMCs, suggesting they may be beneficial targets for suppressing restenosis. It has been reported that HDAC activates the Notch signaling pathway via PI3K/AKT, JNK, and p38 MAPK pathways, promoting VSMC differentiation and inhibiting phenotypic transformation, thereby helping prevent intimal thickening and restenosis [355]. SIRT1 overexpression significantly inhibits neointima formation following carotid artery ligation or wire injury in vivo and significantly suppresses VSMC proliferation and migration in vitro. Through its deacetylase activity, SIRT1 specifically reduces the expression of Cyclin D1, inducing G1/S cell cycle arrest. SIRT1 also inhibits extracellular matrix degradation by reducing MMP9 expression, thereby attenuating VSMC migratory capacity. Knocking down SIRT1 has the opposite effect, indicating its beneficial role in reducing vascular restenosis [356]. Furthermore, SIRT1 and SIRT3 reportedly inhibit VSMC proliferation, migration, and neointima formation in Ang II-induced vascular remodeling [357, 358].

Trichostatin A (TSA), an HDAC inhibitor, enhances AKT activity and upregulates the proliferation and migration of VSMCs by reducing thioredoxin 1 expression, suggesting HDAC inhibition might be detrimental in this context [359]. Conversely, other reports indicate TSA prevents VSMC proliferation and neointimal hyperplasia induced by rat carotid artery balloon injury by upregulating KLF4 expression [360]. These discrepancies could result from different TSA dosages or the distinct roles of specific HDAC subtypes in different models. Targeting specific HDAC subtypes holds promise for treating vascular restenosis (Fig. 5). However, given the dual role of HDACs—potentially promoting VSMC phenotypic transformation and restenosis, or participating in their inhibition—a more precise understanding of the functions and mechanisms of individual HDAC subtypes is crucial. This understanding is essential for developing highly selective HDAC inhibitors or agonists for the treatment of vascular restenosis.

##### Interferon regulatory factor (IRF)

Traditionally known for mediating cellular interferon and immune responses, IRF9 is now recognized as a key player in vascular injury pathology. Studies have shown that IRF9 is upregulated by vascular injury and its knockout inhibits the proliferation and migration of VSMCs and alleviates post-injury intimal thickening. Conversely, IRF9 overexpression exacerbates arterial narrowing through the "IRF9-SIRT1" axis [361]. Specifically, PDGFR-β signaling inhibits SIRT1 expression through IRF9, thereby activating NF-κB signaling and triggering VSMC phenotypic transformation [362]. Furthermore, knockdown of miRNA-17 promotes VSMC proliferation and migration by upregulating IRF9 expression [363]. As a vascular injury-responsive molecule, IRF9 is a potential biomarker for vascular restenosis. Inhibiting IRF9 function (genetically or pharmacologically) could inhibit neointima formation, positioning IRF9 as a potential therapeutic target for preventing vascular restenosis. Interestingly, IRF7 exerts an effect opposite to that of IRF9. Studies have revealed that IRF7 acts as a key regulatory molecule that inhibits neointima formation. Its unique mechanism involves specific protein–protein interactions: IRF7 finely modulates both the transcriptional activity of activating transcription factor 3 (ATF3) and the functional role of PCNA during DNA replication. Through these interactions, IRF7 blocks VSMC cell cycle progression, inhibits cell proliferation, and ultimately reduces neointima formation. Conversely, the absence or downregulation of IRF7 exacerbates these pathological processes [364, 365]. Therefore, IRF7 represents a promising therapeutic target for restraining vascular intimal thickening and restenosis. Members of the IRF family exert counteracting roles, fine-tuning the vascular injury response (Fig. 5). Each member offers a distinct potential pathway for treating vascular restenosis. However, in the process of drug development, it remains highly challenging to identify safe methods that can specifically inhibit one subtype or activate another.

The above dual-role targets serve as complex hub nodes in regulating neointimal hyperplasia due to the dual, and often opposing, functions of their family members. Their advantage lies in the depth of research into their regulatory mechanisms, suggesting considerable theoretical potential for therapeutic intervention. However, significant and challenging drawbacks cannot be overlooked: the antagonistic functions of different isoforms within the same family mean that non-specific broad-spectrum inhibitors or agonists may lead to off-target effects, mutual functional cancellation, or even harmful outcomes. Consequently, the primary challenge is achieving precise, isoform-specific or pathway-specific intervention, which imposes stringent demands on drug design and therapeutic strategy.

### Targeting EC repair

#### Impair EC repair

##### miRNAs

In addition to the above miRNAs targeting VSMCs to promote or inhibit neointimal hyperplasia discussed previously, Ciro Indolf et al*.* [275] also summarized miRNAs targeting ECs, such as miR-17/92a and miR-16 (Table 1). The miR-17/92a functions as a negative regulator of blood vessel growth. Its inhibition enhances EC proliferation and migration, promoting angiogenesis and re-endothelialization, thereby facilitating the repair of damaged blood vessels. Key targets of miR-17/92a include ITGA5 (Integrin, Alpha), SIRT1, KLF2, KLF4, and eNOS. The miR-16 inhibits EC proliferation and migration, negatively impacting endothelial repair. It achieves this by suppressing Rho GDP dissociation inhibitor α (RhoGDIα), VEGF, VEGF receptor 2 (VEGFR2), and FGFR1. Elevated levels of miR-16 are closely associated with an increased risk of limb amputation and restenosis in patients with severe limb ischemia (Fig. 2).

#### Promote Re-endothelialization

##### VEGF

VEGF is a homodimeric glycoprotein that plays a pivotal role in angiogenesis and vascular remodeling. Its mechanisms include inhibiting VSMC proliferation and enhancing EC survival, etc. [176, 366]. However, the effect of VEGF signaling on intimal hyperplasia remains controversial [366, 367]. Most reported cases of VEGF-mediated inhibition of restenosis are attributed to its promotion of re-endothelialization [368]. For instance, a VEGF/paclitaxel nano-coating facilitates early vascular endothelial healing and inhibits VSMC proliferation through the sequential release of VEGF and paclitaxel, leading to significant inhibition of ISR [369]. In porcine models of vascular restenosis, eluting stents coated with VEGF-secreting stem cells or VEGF-loaded adenovirus have been shown to reduce ISR and promote arterial re-endothelialization [370, 371]. Similarly, in rabbit balloon injury models, magnetic nanospheres or poly (lactic-co-glycolic acid) nanoparticles overexpressing VEGF alleviated intimal hyperplasia and restenosis in balloon-injured arteries [372, 373]. Clinical data indicate that serum VEGF levels are significantly lower in patients with late ISR compared to those without ISR, and a negative correlation exists between serum VEGF levels and the occurrence of late ISR [374]. Recent studies report that VEGF binding to Semaphorin 4D (SEMA4D) induces metabolic remodeling of cells, enhances endothelialization, and thereby inhibits neointimal hyperplasia and vascular restenosis [375].

In addition to its roles in promoting re-endothelialization and improving intimal function, early studies indicated that VEGF and its receptor activity can effectively reduce the expression of inflammatory factors and attenuate inflammatory responses, thereby mitigating neointima formation [376]. Moreover, VEGF subtly regulates VSMC behavior. On one hand, it indirectly facilitates a microenvironment conducive to VSMC migration and proliferation by promoting angiogenesis. On the other hand, VEGF may also restrain excessive VSMC proliferation by suppressing specific proliferative signaling pathways, thereby helping maintain vascular structural integrity. However, under certain conditions, VEGF overexpression can trigger phenotypic switching in VSMCs, enhancing their migratory and proliferative capacity and thereby contributing to neointimal thickening. Simultaneously, VEGF plays a key role in extracellular matrix (ECM) remodeling. By regulating the expression and activity of MMPs, VEGF influences ECM degradation and reconstruction, facilitating VSMC migration and accelerating neointimal thickening and the progression of vascular lesions [221]. More complex still, VEGF interacts with multiple other signaling pathways—such as TGF-β/smads and Hippo-YAP/TAZ—creating interconnected regulatory networks that amplify its effects on vascular remodeling and intimal thickening. These interactions are particularly significant in response to vascular injury or pathological stimuli, highlighting the multifaceted role of VEGF [176].

In recent years, researchers have explored dual-targeting strategies aimed at simultaneously enhancing endothelial function and inhibiting VSMC proliferation to prevent intimal thickening and restenosis. For example, microfluidic electrospinning has been used to develop coatings co-loaded with VEGF and rapamycin, which promote endothelial recovery while suppressing VSMC proliferation, resulting in excellent antithrombotic and anti-ISR outcomes [377]. Other researchers have designed stents with polyphenol-polyamine surface modifications that leverage VEGF’s biological functions to selectively enhance EC proliferation and migration while inhibiting VSMC activity [378]. Additionally, dual-targeting nanoparticles that promote VEGF expression while inhibiting ERK2 have been developed to enhance the proliferation and migration of ECs while restricting VSMC proliferation and migration, offering a dual-therapy approach to mitigate vascular restenosis [379].

In addition to the aforementioned studies suggesting that VEGF itself can inhibit intimal thickening and vascular restenosis, some reports present conflicting evidence, indicating that VEGF may potentially accelerate restenosis and atherosclerosis. For instance, application of VEGF and FGF2 to the vascular adventitia has been found to exacerbate restenosis [380]. In a rabbit carotid artery model of intimal hyperplasia, efficient expression of VEGF-A and VEGF-D in the adventitia led to arterial thickening [381]. Elevated VEGF expression has also been detected in internal thoracic artery grafts, while significant levels of VEGFR-3 and caveolin 3 (CAV3) were observed in medial SMC of saphenous vein grafts, implicating both VEGF and CAV3 in early restenosis processes [382]. Furthermore, significantly elevated plasma levels of VEGF after PCI have been reported to correlate positively with the incidence of ISR, suggesting enhanced inflammatory activity at the stent site [383].

**Fig. 6:**
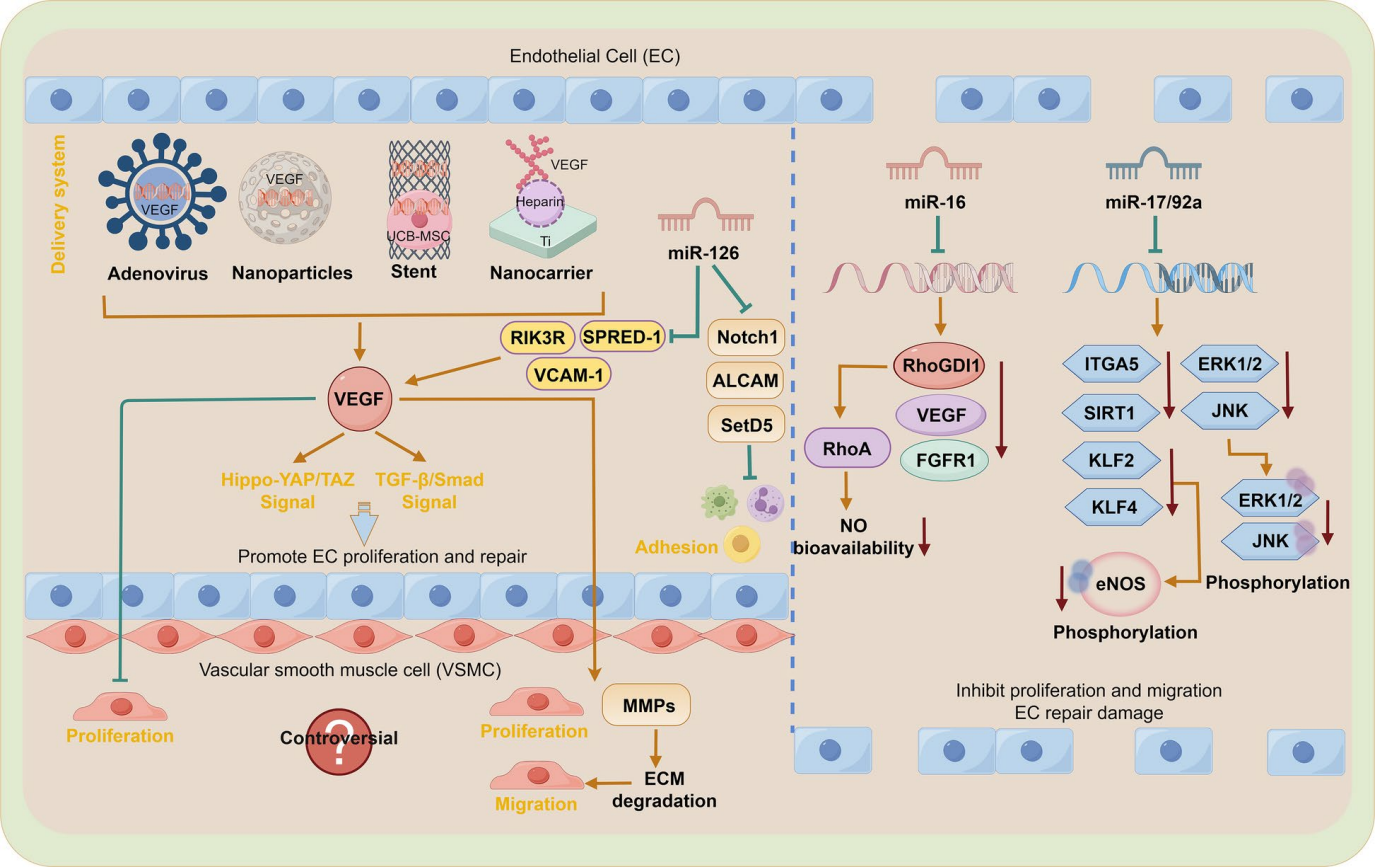
Molecular regulatory network targeting EC repair. VEGF promotes EC proliferation and repair through multiple signaling pathways (including Hippo and TGF-β), while also modulating the phenotype of VSMCs. However, the effect of VEGF on VSMCs remains controversial, with some studies reporting a pro‑proliferative role and others suggesting it inhibits VSMC phenotypic switching. MiRNAs play important roles in regulating EC proliferation. MiR‑126 promotes VEGF expression and EC repair by inhibiting signals such as PI3KR3 and SPRED‑1, while also reducing immune cell adhesion by suppressing pathways including Notch1. MiR‑16 impairs endothelial repair and NO synthesis by inhibiting the expression of genes such as RhoGDI1, VEGF, and FGFR1. MiR‑17/92a reduces EC proliferation, migration, and repair by suppressing ERK and JNK signaling activation, as well as the expression of KLF, ITGA5, and SIRT1. Created in Figdraw.com

In summary, although the majority of studies support a beneficial role of VEGF in preventing vascular restenosis, a minority of reports indicate adverse effects (Fig. 6). The relationship between VEGF and intimal thickening or restenosis thus remains complex and warrants further investigation. VEGF may exert distinct—and sometimes opposing—effects within the intima, media, and adventitia of blood vessels. Its overall impact on vascular restenosis is likely determined by the integrated outcome of these layer-specific actions.

##### miRNAs

In a review by Ciro Indolfi et al*.*, miR-126—which targets ECs and promotes re-endothelialization—was summarized (Fig. 2). miR-126 modulates VEGF and TNF-α signaling pathways through inhibition of sprouting-related proteins such as SPRED-1, phosphoinositide-3-kinase regulatory subunit (PIK3R), and VCAM-1, thus promoting EC proliferation and re-endothelialization. Additionally, miR-126 attenuates leukocyte adhesion and recruitment to ECs by suppressing the Notch1 pathway, leukocyte cell adhesion molecule (ALCAM), and SET domain-containing 5 (SetD5) [275]. Analogous to the ceRNA networks in VSMCs, the function of pro-endothelial miR-126 is also subject to regulation by LncRNAs and circRNAs. For instance, LncRNA NEAT1 has been reported to act as ceRNA for miR-126, thereby modulating EC function [384]. While direct evidence in the context of vascular restenosis is still emerging, this represents a promising area for future investigation, where targeting upstream regulators of miR-126 could be harnessed to promote re-endothelialization and prevent restenosis.

In research on the prevention and treatment of vascular restenosis, several key molecular targets have drawn attention due to their specific regulatory effects on EC function. Among these, miR‑17/92a and miR‑16 primarily exert negative regulatory roles: miR‑17/92a impedes EC proliferation and vascular repair by targeting and suppressing molecules such as ITGA5 and SIRT1, while miR‑16 hinders EC repair through inhibition of pathways involving RhoGDI α and VEGF. Elevated expression of these miRNAs may be closely associated with adverse clinical outcomes (Fig. 6). In contrast, miR‑126 and VEGF act as core factors promoting re‑endothelialization. MiR‑126 enhances EC function and reduces leukocyte adhesion by modulating VEGF and inflammatory pathways (e.g., SPRED‑1, VCAM‑1). VEGF not only directly promotes EC survival and migration but also suppresses excessive VSMC proliferation; however, its effects are context‑dependent and may under certain pathological conditions contribute to inflammation or intimal thickening (Fig. 6). The central translational value of these targets lies in their potential to enable precise interventional strategies for vascular repair. On one hand, modulating specific miRNAs—such as inhibiting miR‑16 or upregulating miR‑126—could directionally improve endothelial repair. On the other hand, VEGF‑based delivery systems (e.g., nanocoated or gene‑coated stents) or dual‑target strategies combining VEGF with anti‑proliferative agents hold promise as innovative approaches to promote functional healing of injured vessels and reduce the risk of restenosis.

### Targeting both VSMC phenotypic switching and EC function

#### PERK

**Fig. 7:**
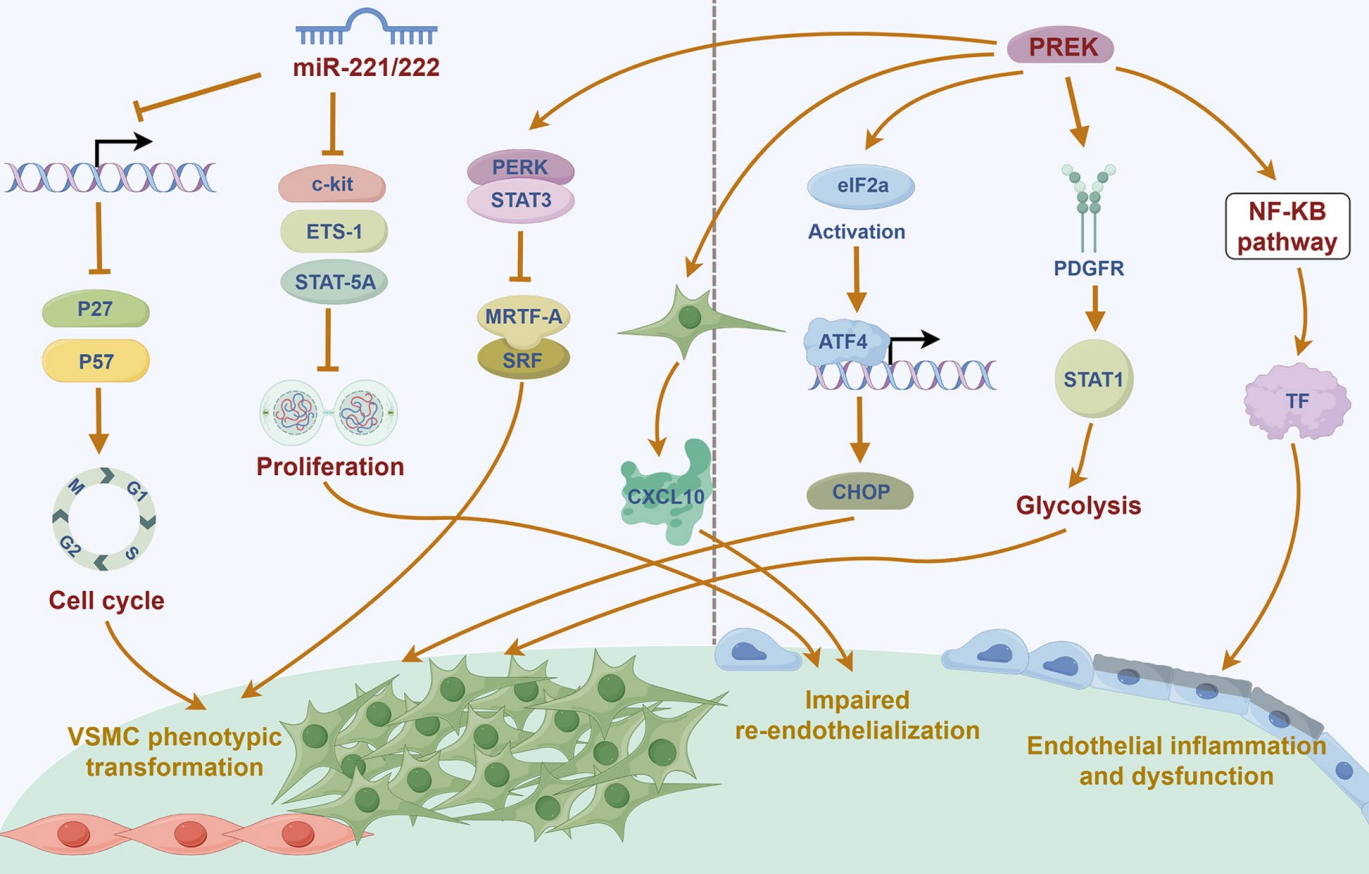
Regulatory network of molecular targets that simultaneously target both VSMC phenotypic switching and EC repair. PERK and miR‑221/222 coordinately regulate VSMC phenotypic switching and EC repair at multiple levels. PERK mediates endothelial inflammation and impairs re‑endothelialization by activating NF‑κB signaling and inducing CXCL10 secretion from VSMCs. Concurrently, PERK drives VSMC proliferation and migration by activating STAT1 and the PERK‑STAT3 axis, as well as by promoting CHOP expression. MiR‑221/222 promotes cell cycle progression by suppressing p27 and p57, thereby mediating VSMC phenotypic switching. In ECs, miR‑221/222 inhibits EC proliferation and re‑endothelialization by suppressing the expression of STAT5A, ETS‑1, and c‑kit. Created in Figdraw.com

While DESs prevent neointimal SMC proliferation, they can exacerbate EC dysfunction and thrombosis. Identifying common targets that simultaneously regulate both VSMCs and ECs has been challenging. Recent studies highlight PERK, a component of the unfolded protein response (UPR) activated under ER stress, as such a target (Fig. 7). PERK plays a crucial role in protein synthesis, cell stress, neointimal formation, and ER-plasma membrane interactions [385]. In rat balloon injury models, PERK protein complexes with STAT3 and MRTF-A. MRTF-A activates the transcription factor SRF, which controls SMC contractile gene expression, driving the phenotypic transformation of VSMCs. PERK inhibitors block this process [386]. In another thrombosis model, PERK promoted the expression of tissue factor (TF, a potent pro-thrombotic factor) through the NF-κB (p65) pathway, thereby exacerbating endothelial inflammation and dysfunction. Pretreatment with a PERK inhibitor abolished this effect and alleviated endothelial dysfunction [386]. This finding supports future PERK-targeted intervention strategies to achieve dual inhibition of VSMC phenotypic transformation and EC dysfunction. Additionally, other studies demonstrate that PERK inhibitor treatment or PERK knockdown regulates VSMC phenotypes and paracrine factor expression, subsequently influencing adjacent ECs. This accelerates re-endothelialization and promotes rapid vascular repair following rat angioplasty [387]. PERK also induces cerebrovascular remodeling by upregulating the PERK-eukaryotic initiation factor 2α (eIF2α)-activating transcription factor 4 (ATF4) signaling axis, facilitating the phenotypic transformation of cerebrovascular VSMCs from a contractile to a synthetic type [388]. In pulmonary artery VSMCs, PERK activation reduces levels of the anti-apoptotic miR124-3p through the UPR pathway, enhancing VSMC viability, proliferation, and glycolysis. Conversely, PERK deficiency inhibits the PDGFRβ-STAT1 signaling pathway and glycolysis, thereby improving pulmonary vascular remodeling [389]. Collectively, these findings indicate that targeted PERK inhibition represents a novel approach to regulate VSMC proliferation and improve the prognosis of vascular diseases (Table 3).

#### miR-221/222

miR-221/222 exerts distinctly opposite effects in ECs and VSMCs (Fig. 2). miR-221/222 promotes VSMC proliferation and phenotypic transformation by suppressing the target genes p27 (Kip1) and p57 (Kip2) (Fig. 7). In contrast, within ECs, miR-221/222 targets the stem cell marker c-kit and modulates ICAM-1, erythroblast transformation specific 1 (ETS-1), and STAT5A (Fig. 7). This inhibits EC proliferation, migration, inflammatory responses, and neovascularization, ultimately impairing re-endothelialization and vascular intimal repair [275].

#### METTL3

N^6^-methyladenosine (m^6^A), the most prevalent internal RNA modification in mammalian mRNAs, plays a crucial role in cardiovascular diseases, including myocardial hypertrophy, ischemic heart disease, vascular calcification, and restenosis. METTL3 is a key transferase for m^6^A modification. Recent studies found that oscillatory stress (OS) upregulates METTL3, leading to m^6^A RNA hypermethylation, increased NF-κB p65 Ser536 phosphorylation, and enhanced monocyte adhesion. Concurrently, METTL3 upregulates NLRP1 (NACHT, LRR, and PYD domain-containing protein 1) transcripts while downregulating KLF4 transcripts, inducing an intimal inflammatory response [390]. In addition, under hypoxic conditions, METTL3 mRNA and protein are abnormally upregulated in pulmonary artery SMCs (PASMCs). This mediates m^6^A modification of phosphatase and tensin homolog (PTEN) mRNA. The m^6^A-binding protein YTHDF2 (YTH N^6^-methyladenosine RNA-binding protein F2) recognizes the modified PTEN mRNA, promoting its degradation. This degradation activates the PI3K/AKT signaling pathway, driving excessive PASMC proliferation. Therefore, the METTL3/YTHDF2/PTEN axis represents a novel therapeutic target for inhibiting abnormal vascular thickening [391]. Further studies revealed that METTL3 promotes DiGeorge syndrome critical region gene 8 (DGCR8) binding to pri-miR-375, increasing the expression of miR-375-3p. MiR-375-3p, in turn, targets and inhibits the transcription of 3-Phosphoinositide-dependent protein kinase 1 (PDK1), thereby promoting the phenotypic transformation of VSMCs [392].

**Fig. 8:**
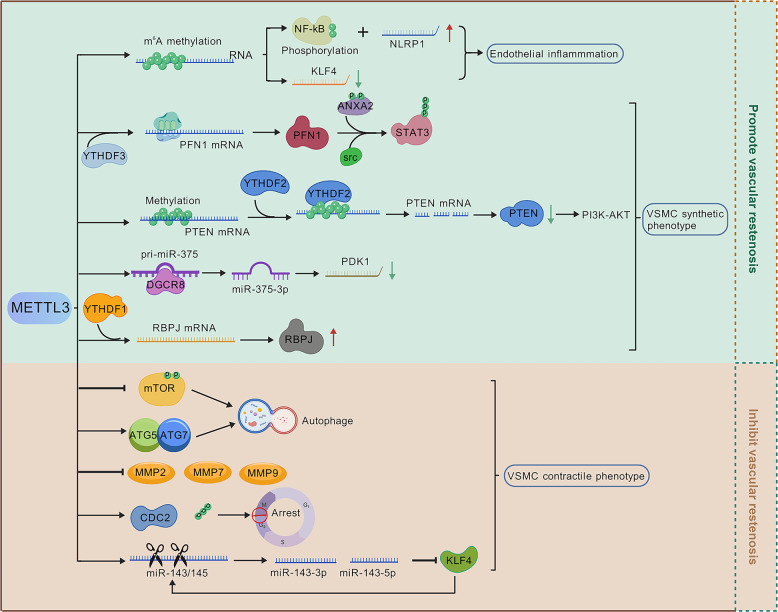
METTL3 plays a dual role in either promoting or inhibiting vascular restenosis. On one hand, it facilitates vascular intimal inflammation by inducing m^6^A RNA hypermethylation, which triggers NF-κB p65 Ser536 phosphorylation, upregulates NLRP1 transcripts, and downregulates KLF4 transcripts. METTL3, in conjunction with YTHDF3, enhances the translation efficiency of PFN1 in an N^6^-methyladenosine-dependent manner. PFN1 then promotes STAT3 phosphorylation by recruiting Src and phosphorylated ANXA2, thereby inducing VSMC phenotypic switching and neointimal hyperplasia. Additionally, METTL3 mediates m^6^A modification of PTEN mRNA, which is recognized by YTHDF2, leading to PTEN degradation, subsequent activation of the PI3K/AKT signaling pathway, and excessive VSMC proliferation. METTL3 also promotes DGCR8 binding to pri-miR-375, increasing miR-375-3p expression; this miRNA targets and inhibits PDK1 transcription, further accelerating VSMC phenotypic switching. Furthermore, METTL3 and YTHDF1 regulate RBPJ mRNA expression in an m^6^A-dependent manner, thereby promoting VSMC proliferation and migration. On the other hand, METTL3 can suppress the synthetic phenotype of VSMCs by promoting autophagosome formation through upregulation of ATG5 and ATG7 and inhibition of mTOR phosphorylation. It inhibits VSMC migration by reducing the protein levels of MMP2, MMP7, and MMP9, and suppresses VSMC proliferation by inactivating CDC2 via phosphorylation, thereby inducing G2/M cell cycle arrest. Moreover, METTL3 facilitates the processing of pri-miR-143/145, leading to increased expression of miR-143-3p and miR-145-5p. MiR-145-5p targets and inhibits KLF4, which in turn promotes transcription of the miR-143/145 cluster, forming a positive feedback loop that maintains the contractile phenotype of VSMCs. Created in Figdraw.com

Recent reports indicate that METTL3 participates in VSMC proliferation and migration through multiple pathways (Fig. 8). METTL3 and YTHDF3 enhance the translation efficiency of profilin-1 (PFN1) in an m^6^A-dependent manner. Subsequently, PFN1 interacts with phosphorylated ANXA2 (Annexin A2) by recruiting Src (Non-receptor tyrosine kinase), promoting STAT3 phosphorylation. This cascade induces the phenotypic transformation of VSMCs and neointimal hyperplasia [393]. Additionally, research groups report that METTL3 and YTHDF1 regulate recombination signal binding protein for immunoglobulin kappa J region (RBPJ) mRNA expression in an m^6^A-dependent manner, promoting PDGF-BB- or hypoxia-stimulated proliferation and migration of PASMCs [394].

Interestingly, some studies present the opposing view, suggesting that METTL3 overexpression can inhibit excessive VSMC proliferation (Fig. 8). For instance, Ze-Min Fang et al*.* found that knocking down METTL3 actually promoted the proliferation, migration, and synthetic phenotype of HASMCs. Conversely, METTL3 overexpression arrested cells at the G2/M checkpoint and inactivated cell division cycle 2 (CDC2) via phosphorylation, thus inhibiting HASMC proliferation. Furthermore, METTL3 overexpression reduced the protein levels of MMP2, MMP7, and MMP9, and promoted autophagosome formation by upregulating autophagy-associated 5 (ATG5) and ATG7 expression, thereby inhibiting the synthetic phenotype of HASMCs [395]. Another study from the same group demonstrated that METTL3 overexpression significantly inhibits mTOR phosphorylation, activates autophagosome formation through the mTOR pathway, and suppresses VSMC proliferation [396]. Recent studies indicate that METTL3-mediated m^6^A modification promotes the processing of miR-143/145 precursors, leading to increased miR-143-3p and miR-145-5p levels. MiR-145-5p targets and inhibits KLF4, which in turn promotes the transcription of miR-143/145, establishing a positive feedback loop. This loop continuously promotes contractile marker gene expression, protecting the contractile phenotype of PASMC [397]. These findings contradict the view that METTL3 promotes VSMC phenotypic switching. This inconsistency may stem from differences in model systems (e.g., in vitro vs. in vivo) or variations in experimental approaches.

## Summary

This review systematically delineates the pathophysiological mechanisms and molecular regulatory networks underlying vascular restenosis, with a particular focus on three core elements: endothelial injury, VSMC phenotypic switching, and inflammatory immune responses. It further explores the signaling network level, elucidating the synergistic regulatory roles of key pathways such as PI3K/AKT, MAPK, and Wnt/β-catenin, as well as epigenetic modifications. Building on this foundation, the review innovatively constructs a "molecular target atlas," systematically categorizing therapeutic targets into several functional classes: pro-proliferative/migratory, anti-proliferative/migratory, and dual-function types targeting SMCs; endothelial repair-related targets; and dual-action regulators (e.g., PERK, miR-221/222) that simultaneously modulate both cell types. Through this hierarchical organization and critical evaluation, the review highlights the evolving research paradigm—from a focus on individual molecules toward an integrated understanding of network regulation—thereby providing a systematic reference framework for future basic research and clinical translation.

Currently, clinical strategies for preventing and treating vascular restenosis mainly fall into two categories: systemic pharmacological therapy and local drug delivery systems. Systemic treatment is primarily represented by statins, antiplatelet agents, and angiotensin-converting enzyme inhibitors, while local delivery relies mainly on drugs such as paclitaxel, SRL, and their derivatives, which are applied locally via coatings or elution from devices [398, 399]. However, existing drugs still face limitations such as significant cytotoxicity, potential immunosuppressive effects, and insufficient target selectivity. Given that neointima formation is the final common pathway of restenosis, dissecting the complex, multi-layered regulatory networks that govern this process is essential for identifying novel, clinically viable therapeutic targets. Significant progress has been made in understanding the mechanisms underlying neointima formation. Studies identifying key molecules and signaling pathways involved in this process have revealed numerous potential therapeutic targets. Several drugs developed based on these targets have entered clinical trials, showing promising therapeutic effects and application prospects. Insights into the mechanisms of neointima formation allow clinicians to design comprehensive treatment strategies. For instance, stent design and coating materials can be optimized to enhance EC repair and coverage while reducing VSMC proliferation and migration, thereby decreasing the incidence of ISR. DES coated with anti-proliferative agents on metal platforms can effectively suppress VSMCs activity, limit neointima formation, and improve patient outcomes.

The pathological mechanism of vascular restenosis involves a multi-level, network-like regulatory process encompassing ECs, VSMCs, the adventitia, and immune cells. The corresponding intervention targets include those targeting VSMC phenotypic switching (e.g., FGF10, LSD1, CGRP), EC repair (e.g., VEGF, miR-16), and dual-targeting agents (e.g., PERK, miR-221/222). Most of these targets influence neointimal hyperplasia and vascular restenosis by regulating VSMCs phenotypic transformation. Notably, different subtypes of the same target may have opposing effects. For example, KLF4 and KLF5 promote neointima formation, whereas KLF15 inhibits VSMC phenotypic transformation and reduces neointima formation. Several HDAC isoforms—including HDAC1, 2, 3, 4, 6, and 7—contribute to vascular restenosis. While HDAC inhibitors can attenuate vascular intimal thickening, there are also reports that the HDAC inhibitor TSA may promote VSMC phenotypic transformation. Similarly, IRF9 mediates vascular restenosis, while IRF7 inhibits neointima formation. Therefore, clarifying the functional specificity of different subtypes is essential for developing subtype-selective inhibitors or agonists. The targets do not function in isolation; rather, they interact through epigenetic modifications, cell cycle progression, and inflammatory signaling, forming an intricate regulatory network. For instance, adventitia-derived FGF10 drives VSMC phenotypic switching via paracrine activation of the MAPK/PI3K/AKT pathway within VSMCs. Similarly, within VSMCs, molecules such as CGRP, MLK3, and PRH S163C:S177C coordinately regulate the contractile phenotype through distinct signaling mechanisms. Some targets exhibit context-dependent effects. For instance, SIRT7 and METTL3 have been reported both to promote and alleviate vascular restenosis in different studies. These dual-role targets may function differently across experimental models and might not be specific to restenosis. Certain targets mediate intimal thickening and restenosis by acting on both ECs and VSMCs. For example, PERK promotes VSMC proliferation via pathways such as the STAT3/MRTF-A axis, exacerbates endothelial inflammation and dysfunction through NF-κB, and induces VSMC secretion of the chemokine CXCL10, which hinders re‑endothelialization. This multifaceted role makes PERK an ideal target for intervening in restenosis. Inhibiting PERK may simultaneously alleviate excessive VSMC proliferation, reduce endothelial inflammation, and promote re‑endothelialization, thereby suppressing the restenosis process in multiple dimensions. Notably, PERK inhibitors have already been extensively studied in preclinical research for cancer and metabolic diseases [400–402].

From a drug development perspective, although some targets (e.g., CGRP, PERK) show significant promise due to their ability to intervene in multiple pathological processes simultaneously, three major challenges remain. First, many key targets (e.g., KLFs, HDACs) belong to families whose members exert antagonistic functions, necessitating the development of isoform‑ or pathway‑specific inhibitors to avoid off‑target effects. Second, therapeutic RNAs (e.g., miR‑126, miR‑22) suffer from low delivery efficiency and poor stability in vivo, requiring localized delivery systems such as nanocarriers or stent coatings for precise administration. Third, many interventions have a narrow therapeutic window: excessive inhibition of VSMC proliferation may impair injury healing, while overexpression of VEGF may inadvertently promote inflammation or atherosclerosis. Therefore, future breakthroughs will likely depend on integrating systems biology with smart materials to develop spatiotemporally controlled, cell‑specific combination delivery strategies. Coupled with patient stratification using biomarkers (e.g., miR‑93‑5p), this could ultimately shift the treatment paradigm from “single‑target inhibition” toward “holistic remodeling of the vascular microenvironment”.

Another important yet understudied aspect is the role of the vascular adventitia in regulating intimal thickening and restenosis. While current research has predominantly focused on EC injury and VSMC phenotypic transformation, the adventitia—though less conspicuous—plays a critical role in the pathophysiology of vascular restenosis. It is now recognized as an endocrine organ capable of secreting various bioactive molecules that regulate vascular function [403, 404] (Fig. 1). Notably, adventitia-derived FGF10 has been closely linked to intimal hyperplasia and restenosis [258, 259]. However, studies on adventitial mechanisms in restenosis remain limited. Elucidating the specific regulatory functions of the adventitia will improve our understanding of the pathogenesis of vascular restenosis and may reveal novel therapeutic targets, offering new strategies for its prevention and treatment. Furthermore, a healthy lifestyle serves as an irreplaceable foundation for safeguarding cardiovascular health and preventing vascular restenosis. Positive lifestyle habits can effectively improve vascular function, mitigate inflammatory status, stabilize metabolic parameters, and enhance overall quality of life [405, 406].

## Data Availability

The data used to support the findings of this study are available from the corresponding authors upon request.

## Abbreviations

VSMC: Vascular smooth muscle cell
HA-VSMC: Human aortic VSMC
PASMC: Pulmonary artery SMC
EC: Endothelial cell
EPC: Endothelial progenitor cells
CCN5: Cellular communication network factor 5‌
CKD: Chronic kidney disease
AVF: Arteriovenous fistula
IS: Indoxyl sulfate
EMP: Endothelial microparticle
TGF-β: Transforming growth factor-β
Nrf2: Nuclear factor erythroid 2-related factor 2
HO-1: Heme oxygenase 1
CVD: Chronic venous disease
NO: Nitric oxide
mtROS: Mitochondrial ROS
NLRP3: NOD-like receptor protein 3
RvE1: Resolvin E1
HGF: Hepatocyte growth factor
IFI35: Interferon-induced protein 35
iPSC: Induced pluripotent stem cell
PRMT5: Protein arginine methyltransferase 5
SND1: Staphylococcal nuclease domain-containing protein 1
TAZ: Transcriptional co-activator with PDZ-binding motif
Bves: Blood vessel epicardial substance
Dusp1: Dual-specificity protein phosphatase 1
BMPER: Bone morphogenetic protein (BMP) EC precursor-derived regulator
IGF-BP4: Insulin-like growth factor-binding protein 4
NExN: Nexilin
ACE2: Angiotensin-converting enzyme 2
NR1D1: Nuclear receptor subfamily 1 group D member 1
Gas1: Growth arrest-specific 1‌
DLP1: Dynamin-like protein 1
HDL: High-density lipoprotein
Drp1: Dynamin-related protein 1
AKAP1: Mitochondrial protein A-kinase anchor protein 1
SCF: Stem cell factor
PD: Prednisolone
SRL: Sirolimus
MO: Magnolia kobus DC
GA: Ginkgolic acid
TCTN1: Tectonic 1
FSP-1: Fibroblast-specific protein 1
STING: Stimulator of interferon genes
NDRG1: N-myc downstream-regulated gene 1
RvD1: Resolvin D1
GDF-15: Growth differentiation factor 15
CD74: Cluster of differentiation 74
PKB: Protein kinase B
GSK-3β: Glycogen synthase kinase-3β
MCP-1: Monocyte chemoattractant protein-1
sGC: Soluble guanylyl cyclase
CCN1: CCN family member 1
TAK1: TGF-β-activated kinase 1
S1P: Sphingosine-1-phosphate
S1PR1: Sphingosine-1-phosphate receptor 1
SPHK1: Sphingosine kinase 1
GJα1: Gap junction alpha-1 protein
ox-LDL: Oxidized low‑density lipoprotein
HUVEC: Human umbilical vein endothelial cell
MLCK: Myosin light-chain kinase
MLC: Myosin regulatory light chain
ET-1: Endothelin-1
GSDMD: Gasdermin D
Foxm1: Forkhead box M1
CTGF: Connective tissue growth factor
TEAD: Transcriptional enhanced associate domain
Sema3G: Semaphorin 3G
LATS1: Large tumor suppressor kinase 1
PFKFB3: 6‑Phosphofructo‑2‑kinase/fructose‑2, 6‑biphosphatase 3
LLPS: Liquid–liquid phase separation
TET2: Ten–eleven translocation 2
DNMT1: DNA methyltransferase 1
CBP: CREB-binding protein
KDM1A: Lysine-specific demethylase 1A
VEGF: Vascular endothelial growth factor
IL-6: Interleukin-6
LSD1: Lysine-specific demethylase 1
H3K4: Histone 3 lysine 4
PDGF-BB: Platelet-derived growth factor-BB
KLF: Kruppel-like factor
FAM3A: Family with sequence similarity 3
member A; PI3K: Phosphatidylinositol 3-kinase
AKT: Protein kinase B
SM22α: Smooth muscle 22α
ADK: Adenosine kinase
AMP: Adenosine monophosphate
PGK1: Phosphoglycerate kinase 1
USP: Ubiquitin-specific peptidase
UPS: Ubiquitin-proteasome system
HDAC: Histone deacetylase
Skp2: S-phase kinase-associated protein 2
α-SMA: α-Smooth muscle actin
SM-MHC: Smooth muscle myosin heavy chain
MAPK: Mitogen-activated protein kinase
MAP3K5: Mitogen-activated protein kinase kinase kinase 5
MK2: MAPK-activated protein kinase 2
MDM2: Mouse double minute 2
HSP27: Heat shock protein 27
MRTF: Myocardin-related transcription factor
SRF: Serum response factor
CDK: Cyclin-dependent kinase
FOXO1: Forkhead box O1
FOXO3: Forkhead box O3
SIRT: Sirtuin
FGF10: Fibroblast growth factor 10
SHR: Spontaneously hypertensive rat
VAF: Adventitial fibroblast
FGFR: FGF receptor
IRF9: Interferon regulatory factor 9
PDGFR: PDGF receptor
NF-κB: Nuclear factor-κB
NIK: NF-κB-inducing kinase
DOT1L: Telomeric silencing 1-like
CCL5: C-C motif chemokine ligand 5
CXCL10: C-X-C motif chemokine ligand 10
ULK1: Unc-51-like kinase 1
KAT2A: K-acetyltransferase 2A
ADAM22: A disintegrin and metalloprotease 22
ERK: Extracellular signal-regulated kinase
Sox10: Sex-determining region Y (SRY)-related HMG-box gene 10
RGS5: G protein signaling 5
PLC-β: Phospholipase C-β
S-1-P: Sphingosine-1-phosphate
Ang II: Angiotensin II
ROS: Reactive oxygen species
NOX1: NADPH oxidase 1
G6PD: Glucose-6-phosphate dehydrogenase
PPP: Pentose phosphate pathway
Bax: BCL2-associated X
VDAC: Voltage-dependent anion channel
miRNA: MicroRNA
Mfn2: Mitofusin 2
ISR: In-stent restenosis
LncRNA: Long non-coding RNA
RhoA: Ras homolog gene family member A
ROCK: Rho-associated coiled -coil containing protein kinase
STAT3: Signal transducer and activator of transcription 3
ceRNAs: Competing endogenous RNAs
eNOS: Endothelial nitric oxide synthase
RhoGDIα: Rho GDP dissociation inhibitor α
VEGFR: VEGF receptor
ER: Endoplasmic reticulum
PERK: Protein kinase RNA-like ER kinase
UPR: Unfolded protein response
eIF2α: Eukaryotic initiation factor 2α
ATF4: Activating transcription factor 4
ICAM-1: Intercellular adhesion molecule 1
ETS-1: Erythroblast transformation specific 1
METTL3: N6-methyladenosine methyltransferase
m^6^A: N^6^-methyladenosine
OS: Oscillatory stress
NLRP1: NACHT, LRR, and PYD domain-containing protein 1
PTEN: Phosphatase and tensin homolog
YTHDF2: YTH N6-methyladenosine RNA-binding protein F2
DGCR8: DiGeorge syndrome critical region gene 8
PDK1: 3-Phosphoinositide-dependent protein kinase 1
PFN1: Profilin-1
ANXA2: Annexin A2
Src: Non-receptor tyrosine kinase
RBPJ: Recombination signal binding protein for immunoglobulin kappa J region
CDC: Cell division cycle
MMP: Matrix metalloproteinase
ATG: Autophagy-associated protein
PRH: Proline-rich homeodomain
HHEX: Hematopoietically expressed homeobox
JNK: C-Jun N-terminal kinase
TSA: Trichostatin A
MLK3: Mixed-lineage kinase 3
CGRP: Calcitonin gene-related peptide
PKA: Protein kinase A
PKC: Protein kinase C
MSC: Mesenchymal stem cell
PPAR: Peroxisome proliferator-activated receptor
Epc1: Enhancer of polycomb 1
P4HA2: Prolyl 4-hydroxylase alpha-2
YAP1: Yes associated protein 1
Glut10: Glucose transporter 10
TET: Ten-eleven translocation
VitC: Ascorbic acid
DHA: Dehydroascorbic acid
mtDNA: Mitochondrial DNA
RIPK1: Receptor interacting protein kinase 1
EEF1AKMT3: EEF1A lysine methyltransferase 3
MTM: Myotubularin
MTMR7: Myotubularin-related protein 7
mTOR: Mammalian target of rapamycin
mTORC: mTOR complex
MECP2: Methyl-CpG-binding protein 2
EVI1: Ecotropic viral integration site 1
PCI: Percutaneous coronary intervention
HMGB1: High mobility group box 1
circRNAs: Circular RNAs
circMAP3K5: Circular mitogen-activated protein kinase kinase kinase 5
SNHG18: Small nucleolar RNA host gene 18
IRF7: Interferon regulatory factor 7
ATF3: Activating transcription factor 3
PCNA: Proliferating cell nuclear antigen
SEMA4D: Semaphorin 4D
ECM: Extracellular matrix
CAV3: Caveolin 3
TNF-α: Tumor necrosis factor-α
PIK3R: Phosphoinositide-3-kinase regulatory subunit
SPRED-1: Sprouty-related, EVH1 domain-containing protein 1
VCAM-1: Vascular cell adhesion protein 1
ALCAM: Leukocyte cell adhesion molecule
SetD5: SET domain-containing 5
DES: Drug-eluting stent
Myocd: Myocardin
N-Shh: N-terminal Sonic Hedgehog
